# Silver Nanocubes: From Serendipity to Mechanistic Understanding, Rational Synthesis, and Niche Applications

**DOI:** 10.1021/acs.chemmater.3c00472

**Published:** 2023-04-17

**Authors:** Veronica Pawlik, Shan Zhou, Siyu Zhou, Dong Qin, Younan Xia

**Affiliations:** † School of Chemistry and Biochemistry, Georgia Institute of Technology, Atlanta, Georgia 30332, United States; ‡ Department of Nanoscience and Biomedical Engineering, South Dakota School of Mines and Technology, Rapid City, South Dakota 57701, United States; § School of Chemical and Biomolecular Engineering, Georgia Institute of Technology, Atlanta, Georgia 30332, United States; ¶ School of Materials Science and Engineering, Georgia Institute of Technology, Atlanta, Georgia 30332, United States; ⊥ The Wallace H. Coulter Department of Biomedical Engineering, Georgia Institute of Technology and Emory University, Atlanta, Georgia 30332, United States

## Abstract

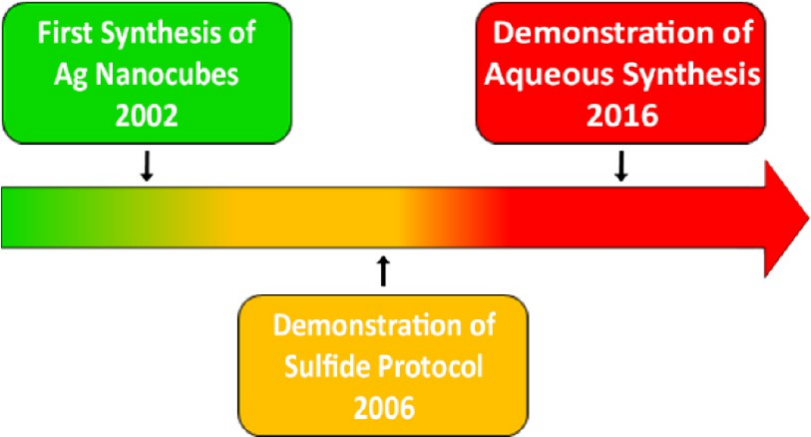

Silver has long been interwoven into human history, and its uses have evolved from currency and jewelry to medicine, information technology, catalysis, and electronics. Within the last century, the development of nanomaterials has further solidified the importance of this element. Despite this long history, there was essentially no mechanistic understanding or experimental control of silver nanocrystal synthesis until about two decades ago. Here we aim to provide an account of the history and development of the colloidal synthesis of silver nanocubes, as well as some of their major applications. We begin with a description of the first accidental synthesis of silver nanocubes that spurred subsequent investigations into each of the individual components of the protocol, revealing piece by piece parts of the mechanistic puzzle. This is followed by a discussion of the various obstacles inherent to the original method alongside mechanistic details developed to optimize the synthetic protocol. Finally, we discuss a range of applications enabled by the plasmonic and catalytic properties of silver nanocubes, including localized surface plasmon resonance, surface-enhanced Raman scattering, metamaterials, and ethylene epoxidation, as well as further derivatization and development of size, shape, composition, and related properties.

## 1 Introduction

Silver (Ag) has become so ubiquitous that it is synonymous with second place. However, its unique properties make it far from inconsequential. More familiar uses of this metal include jewelry and coinage such as silver dollars. Despite this, only an estimated 15% of domestic consumption in 2021 made up these categories.^1^ Instead, nearly 60% of the Ag was used industrially to produce, both directly and indirectly, consumer goods, with notable examples including electronics and pharmaceuticals.^1^ Human history is inextricably interwoven with Ag. Evidence of Ag processing includes not only mining but also refining techniques, such as cupellation, which dates back to as early as the fourth century BC.^2^ Indeed, during this earlier time period, Ag was mainly utilized for its aesthetic and monetary value, as evidenced by numerous archeological finds that reside in museums today.^3^ However, creative uses that take advantage of more than just its metallic qualities did not take long to appear. The writings of Hippocrates indicate that the use of Ag in medicine is as old as the formalized concept of medicine itself, going back to at least 400 BC.^4,5^ Throughout the centuries, it has been used as a treatment for burns, wounds, and epilepsy, as well as in medical equipment. Unlike many other ancient medicinal practices, the apparent “healing” properties of Ag did not let it fall out of vogue. Modern research has revealed that its mode of action involves selective interaction with membrane proteins on microorganisms, resulting in disruption of the proton motive force and cell leakage to make it an extremely potent antimicrobial with low human toxicity.^6,7^ This unique feature led to its extensive use as a primitive type of antibiotic in the 1940s. Even now, the continual rise of antibiotic resistance has, once again, brought Ag to the forefront as an effective alternative.^7^

In addition to its persistent relevance to medicine, other qualities such as high electrical and thermal conductivity, as well as photosensitivity, have further expanded the use of Ag in other long-standing applications such as catalysis and photography.^8−10^ Although waning in use in the digital age, film-based photography dominated the 20th century by offering the capability to form latent image centers upon exposure to light. Key to the operation of a photographic film, silver halide microcrystals embedded in a gelatin matrix can be reduced, when irradiated by light, to generate clusters consisting of 3–5 Ag atoms, which then catalyze the reduction of remaining silver halide for the creation of a visible image.^8^ When used in a more traditional chemical setting, Ag is unique in catalyzing the oxidation of ethylene to ethylene oxide, as well as methanol to formaldehyde.^10^ Both products are vital feedstocks for the plastic industry. As nanotechnology advances, new horizons have also arisen as showcased by many examples that incorporate Ag nanomaterials into electronic devices, display units, solar panels, wearable sensors, and batteries, among others.^11−18^

**Figure 1:**
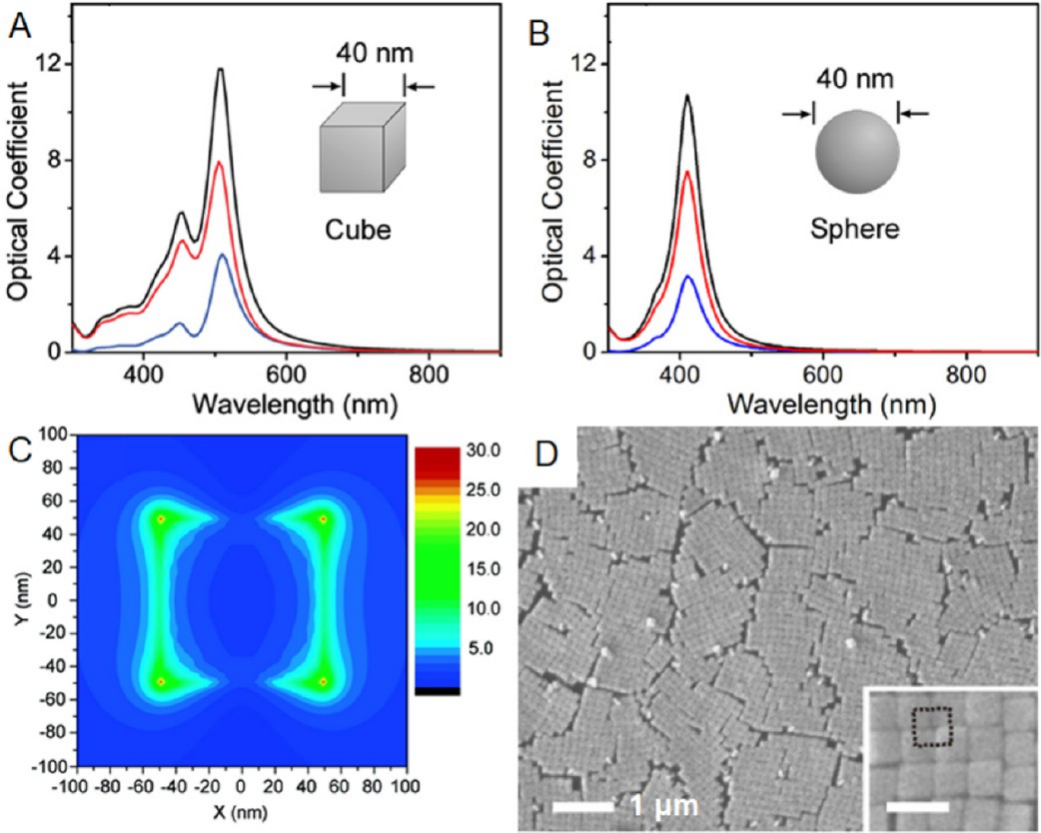
(A, B) The calculated extinction (black), absorption (red), and scattering (blue) spectra of a Ag nanocube (A) and a Ag nanosphere (B) of the same size. (C) Electric field amplitude along the *Z*-axis ([100] direction) when incident light hits the Ag nanocube along the *X*-axis. (D) Scanning electron microscopy (SEM) images of a close-packed array assembled from Ag nanocubes. The scale bar in the inset is 200 nm. (A, B) Reproduced with permission from ref (19). Copyright 2006 American Chemical Society. (C) Reproduced with permission from ref (22). Copyright 2007 American Chemical Society. (D) Reproduced with permission from ref (24). Copyright 2016 American Chemical Society.

Among different forms of Ag nanomaterials, those featuring a cubic shape are particularly attractive for a variety of applications. The cubic shape ensures the prevalence of {100} facets on the surface to maximize their activity and/or selectivity toward particular structure-sensitive reactions. As shown in Figure 1A,B, the cubic shape generates multiple localized surface plasmon resonance (LSPR) peaks, in contrast to its spherical counterpart that only gives one resonance peak.^19−21^ The sharp corners and edges on a cube also cause substantial enhancement in the local electric field, creating “hot spots” instrumental to surface-enhanced Raman scattering (SERS) and related applications (Figure 1C).^22,23^ Moreover, the regular cubic shape lends itself well to self-assembly (Figure 1D), making it possible to take advantage of both individual particles and their ordered arrays for optical applications.^24,25^

While colloidal synthesis of Ag nanocrystals can be traced back to the first report by Lea in 1889,^26^ attaining the cubic shape remained a challenge until 2002.^27^ Crystallized in the face-centered cubic (*fcc*) lattice, the relative surface energies of the three low-index facets decrease in the order of γ_{110}_ > γ_{100}_ > γ_{111}_ in the absence of any surface passivation.^28^ Since the surface of Ag nanocubes is not enclosed by the most stable {111} facets, it is impossible to generate such a morphology under thermodynamic control without introducing a surface capping agent with selectivity toward {100} facets. Further complication arises when multiply twinned seeds are formed in the nucleation step because of the low energy barrier to twinning. This trend also makes it a challenge to fulfill another key requirement for the synthesis of Ag nanocubes, that is, the exclusive creation of single-crystal seeds during the nucleation step and conservation of such a single-crystal structure throughout the growth process. Up until 2002, hundreds of reports on the colloidal synthesis of Ag nanocrystals existed, but all samples were plagued by polydispersity in terms of twin structure, shape, and/or size.^29−33^

## 2 From Serendipity to Mechanistic Understanding

### 2.1 Serendipity

**Figure 2:**
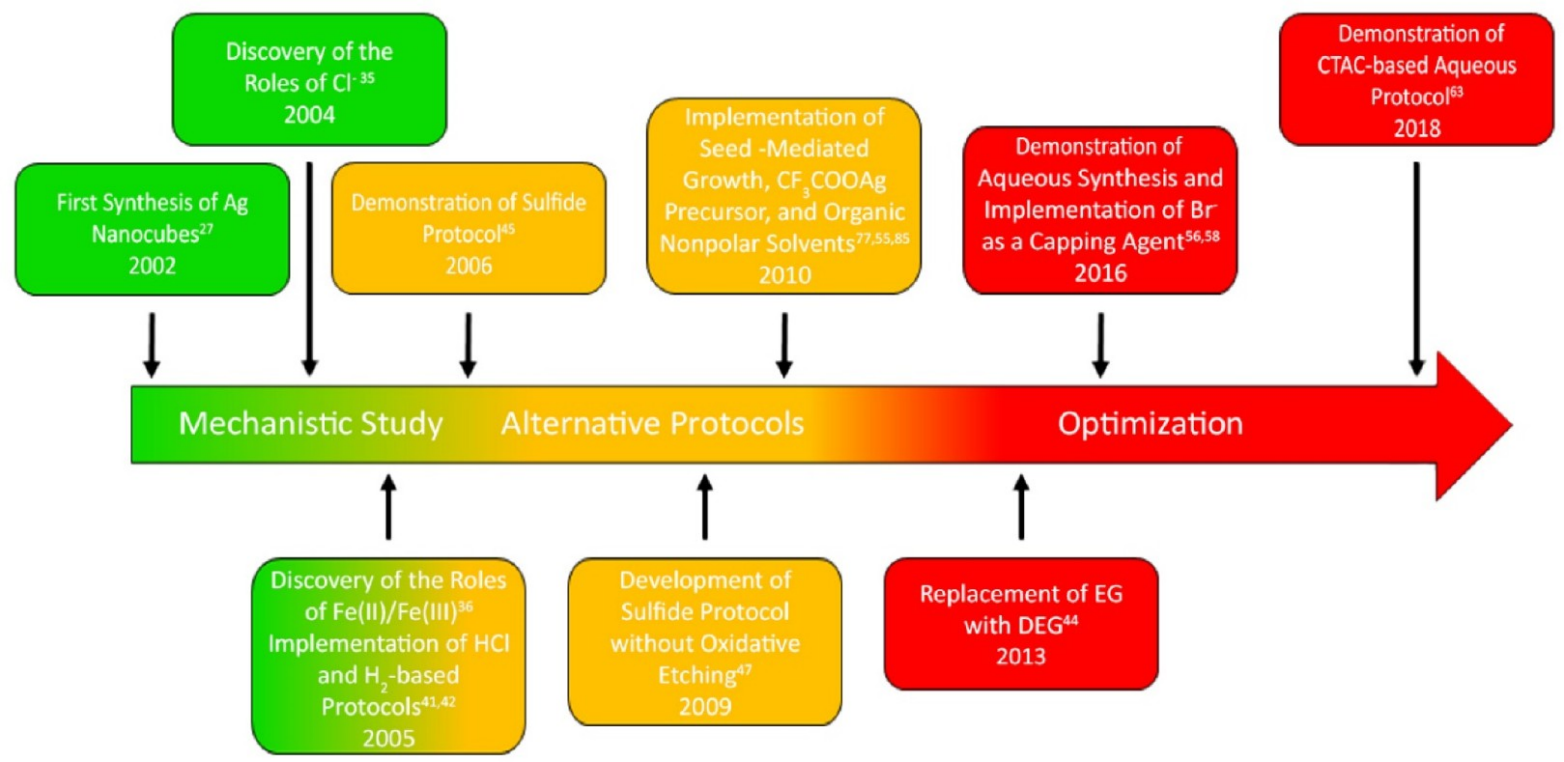
Timeline marking the major developments in colloidal synthesis of Ag nanocubes.

Figure 2 shows a timeline that accounts for our major accomplishments in colloidal synthesis of Ag nanocubes to the present day. Our 2002 paper in *Science* marks the beginning of synthetic control over the shapes taken by Ag nanocrystals, albeit it took almost one decade to elucidate the mechanistic details.^27^ Our original synthesis was based upon the polyol method, in which ethylene glycol (EG) served as both a solvent and a precursor to the reducing agent. It was critical to regulate the reducing power of EG by carefully controlling the reaction temperature. Specifically, nanocubes were obtained in high purity at 160 °C, but irregular nanoparticles tended to appear when the temperature was either reduced to 120 °C or increased to 190 °C. To avoid the formation of Ag nanowires, the AgNO_3_ precursor had to be used at a concentration above 0.1 M. Last but not least, poly(vinylpyrrolidone) (PVP) had to be introduced at a molar ratio of 1.5 between the repeating unit of PVP and AgNO_3_ to selectively passivate and thereby stabilize the Ag{100} facets for the creation of nanocubes. Transmission electron microscopy (TEM) and X-ray diffraction (XRD) results confirmed the formation of uniform Ag nanocubes with an average edge length of 175 nm. The synthesis was also robust enough to produce smaller nanocubes if the concentration of AgNO_3_ and reaction time were slightly modified.

In general, generating single-crystal Ag nanocubes requires the formation of single-crystal seeds in the nucleation step and the preferential expression of {100} facets on the surface during the growth step. As discussed in the Introduction, for an *fcc* crystal such as Ag, the {111} facets have a lower surface energy than the {100} facets, so growth is more favorable along the ⟨100⟩ direction to help eliminate the higher energy facets. To prevent this and thereby obtain a nanocube bounded by the {100} facets, the ratio of growth rates along the ⟨100⟩ and ⟨111⟩ directions must be adjusted to 0.58.^28^ The preferential binding of PVP to the {100} facets can block the surface, decelerating the growth along the corresponding direction. This capping effect was postulated in our original publication.^27^ However, the formation of single-crystal seeds was not discussed, and an explanation of its exclusivity was not attempted either. Our mechanistic understanding was also further obscured by the unclear relationship between PVP and twinning as multiply twinned particles were produced when no or too much PVP was involved. Although individual batches of Ag nanocubes could be prepared with a remarkably high purity, the lack of a mechanistic understanding inevitably led to poor reproducibility between batches due to the variations in chemical reagents and other experimental conditions. The quest for a mechanistic understanding and therefore achievement of reproducible synthesis naturally became the focus of our experimental efforts in the following two decades.

### 2.2 Mechanistic Understanding

The roles of most components in the synthesis were understood and acknowledged in the original nanocube synthesis;^27^ however, the influence of some trace inorganic species was not. While EG was recognized as both a solvent and a precursor to the reducing agent, it also indirectly served an unexpected role. Although long acknowledged, the contamination of EG with inorganic species due to the synthetic and/or storage methods was seldom relevant outside of analytical purposes.^34^ However, in the case of Ag nanocube synthesis, trace amounts of two common contaminants were soon found to hold the key to a successful synthesis.^35,36^

We started to investigate the influence of chloride ions (Cl^–^) in 2004.^35^ After evaporating samples of the as-received EG from various vendors, the remaining salts were analyzed to measure the contents of Cl^–^ ions using ion chromatography. It was determined that EG supplied by J. T. Baker contained the least amount of Cl^–^ and was selected as the appropriate solvent for further experiments. To understand the influence of Cl^–^ on the formation of Ag nanocubes, specific amounts of NaCl or KCl were intentionally introduced into the synthesis. The slow growth of nanocubes was then monitored visually, as well as through UV–vis spectroscopy and TEM imaging.

Over a course of 46 h, four distinct phases were observed for the standard synthesis that involved the use of NaCl at 0.22 mM. Immediately following the injection of the AgNO_3_/EG solution into preheated EG, the reaction mixture turned yellow, indicating the formation of Ag nanoparticles. TEM images taken at this early point showed a mix of small single-crystal particles and a much larger proportion of twinned particles. After 2 h into the synthesis, the solution slowly turned colorless, while a silvery coating was observed on the inner surface of the reaction flask. The corresponding TEM image showed very few particles in the reaction solution, indicating that the as-formed Ag particles had been either dissolved or deposited onto the inner surface of the flask. Importantly, of the remaining particles, a greater proportion was found to be single crystalline in structure. After 7 h into the synthesis, the reaction solution remained colorless, but the silvery coating had mostly disappeared. The fourth stage of the reaction began at *t* = 24 h, when the solution started to turn yellow again with the color constantly growing in intensity for the next 20 h. TEM images taken from the sample obtained at *t* = 44 h into the synthesis showed exclusively single-crystal particles.

**Figure 3:**
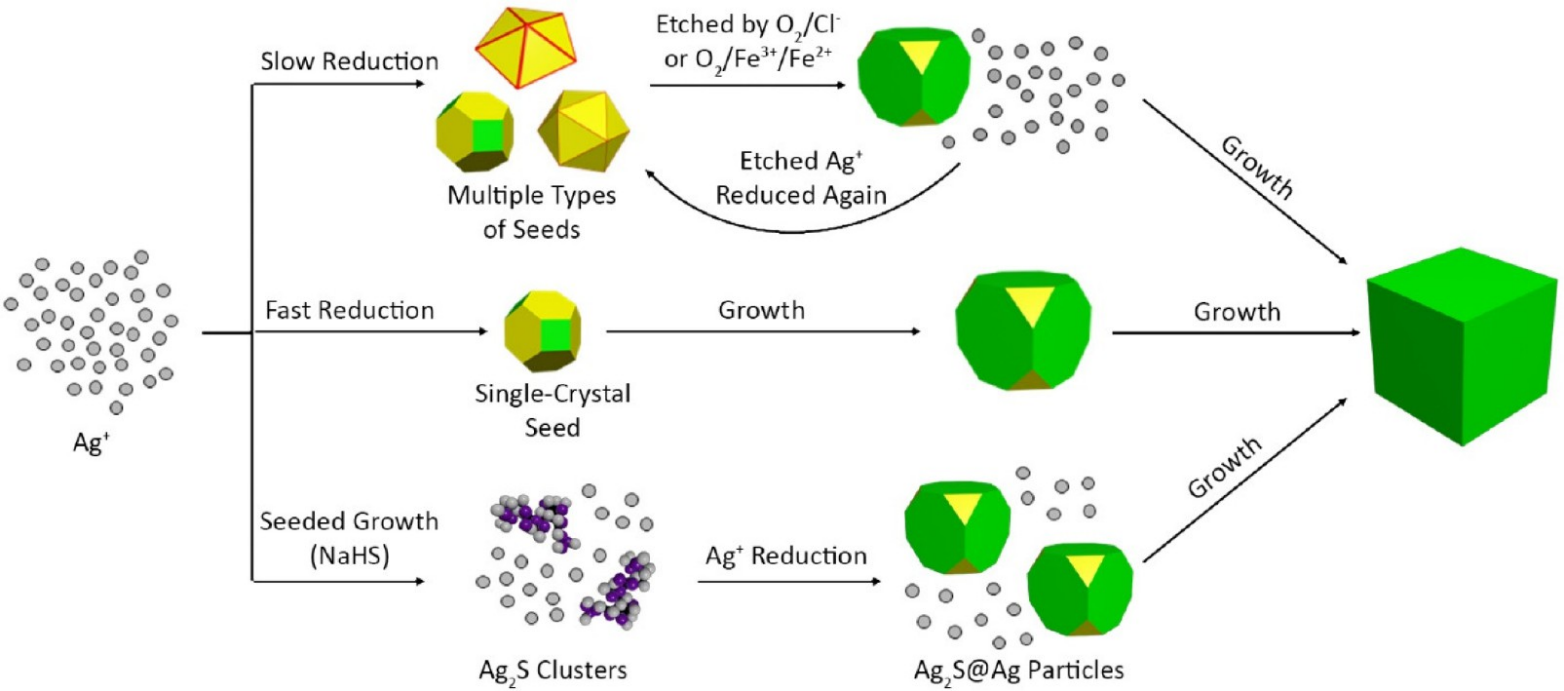
Schematic illustrations of various pathways for the colloidal synthesis of Ag nanocubes.

When a particle is extremely small, both surface and bulk energies make up a significant proportion of the particle’s total energy. Analysis of elastic strain and Gibbs free energy suggests that faceting and twinning exist in a careful balance that creates local minima instead of one global minimum on the potential energy surface.^37,38^ Thus, trading between lower bulk and surface energies can result in twinned particles covered by {111} facets, such as decahedral particles, alongside single-crystal particles covered by {100} facets with a higher surface energy. However, the significant lattice distortions necessary to create decahedral particles also makes them more susceptible to etching by providing higher energy sites for O_2_ to bind to. The appearance, dissolution, and subsequent reappearance of particles alongside the change in crystallinity observed in our synthesis suggested the involvement of such an etching mechanism.^35^ Indeed, when the same experiment was repeated under Ar protection, twinned particles persisted and they could even grow into long penta-twinned nanowires. Altogether, it was concluded that oxidative etching enabled by a combination of O_2_ and Cl^–^ played a pivotal role in ensuring the formation of single-crystal seeds. The Cl^–^ was also proposed to function as a stabilizer for the initially formed Ag seeds by preventing them from aggregating while decelerating the growth rate. Altogether, the mechanism, as described in the top panel of Figure 3, requires a subtle balance between etching and nucleation such that Cl^–^ can slow down growth allowing the dissolved O_2_ to selectively etch away the twinned particles.

Considering that oxidative etching was instrumental to the production of single-crystal seeds, we further investigated the influence of the Fe(III) species given that it is both a common contaminant in EG and a well-known etchant of Ag.^34,36,39,40^ We chose to use Fe(acac)_3_ instead of FeCl_3_ so that the concentrations of Fe(III) and Cl^–^ could be adjusted independently. The role of Cl^–^ was quickly confirmed when syntheses excluding it would result in aggregated, irregular particles. Interestingly, the addition of Fe(acac)_3_ led to two very different results depending on the concentration. When kept below 0.44 μM, Fe(III) ensured the formation of single-crystal Ag nanocubes in a fraction of the time required when only Cl^–^ was used. However, when the concentration exceeded 2.2 μM, long, penta-twinned nanowires were obtained. The use of Fe(III) at low concentrations seemingly increased the rate of oxidative etching while preventing it entirely at high concentrations. To explain these contradicting results, we had to take into account the elevated temperature at which the synthesis was conducted. It was suggested that the Fe(III) species was likely reduced by EG at the high temperature to Fe(II), a species that can no longer etch Ag. Subsequent experiments involving the replacement of Fe(acac)_3_ with Fe(acac)_2_ produced the same results, supporting this argument. Instead of participation as an oxidative etchant, Fe(II) was assumed to be oxidized by O_2_ to generate Fe(III). The generated Fe(III) could then be quickly reduced back to Fe(II) by EG at the high temperature, restarting the cycle and thereby protecting twinned particles. At high concentrations, this mechanism completely prevented etching and helped preserve the twinned particles which could then grow into long nanowires. At sufficiently low concentrations, however, Fe(II) was able to slow down oxidative etching by consuming the dissolved O_2_. As a result, it better preserved single-crystal particles while greatly reducing the duration of time previously required by the first pathway in Figure 3.

Once these studies had elucidated the mechanistic details responsible for the success of our original synthesis in 2002, other components could be added to ensure and even promote the formation of single-crystal seeds. Protons (H^+^) were the first to be added in the form of HCl or H_2_ gas.^22,41,42^ In both cases, H^+^ recombined with the nitrate (NO_3_^–^) from AgNO_3_ to create HNO_3_, which could then act as an additional etchant to O_2_/Cl^–^, slowing down the overall reaction rate while promoting the formation of single-crystal seeds. This process increased the perfection and uniformity of the as-obtained nanocubes. Another strategy that proved effective in exclusively forming single-crystal seeds was to force an extremely fast initial reduction rate (Figure 3, the second pathway).^43^ Manipulating the reaction kinetics can also cause deviations from thermodynamic products, and it has been demonstrated for Ag on occasion, notably when EG is substituted with diethylene glycol (DEG).^44^ Seed-mediated growth involving NaHS or Na_2_S has also been extremely effective (Figure 3, the third pathway).^45−47^ The addition of a trace amount of either NaHS or Na_2_S at a neutral pH generates HS^–^ and H_2_S in the reaction solution. Upon the addition of Ag^+^, Ag_2_S clusters are formed, which can catalyze the further reduction of Ag^+^ by lowering the reduction potential. When combined with an appropriate temperature and concentration of PVP, the inclusion of Ag_2_S clusters can cut the reaction time from more than 40 h down to tens of minutes.

Producing Ag nanocubes through polyol reduction at an elevated temperature is not exclusive, but by far the most common up to this point. Few aqueous syntheses existed, and even fewer were not mediated with preformed seeds. In other words, most of the protocols typically used a polyol method to generate the seeds made of Ag or other metals.^48−54^ Interestingly, PVP is notably absent from most aqueous syntheses. In all the polyol syntheses up to this point, PVP has been proposed to serve as a capping agent for the Ag{100} facets. However, its bulky size can be detrimental to the formation of sharp corners, in particular, for Ag nanocubes with relatively small sizes. Instead, Cl^–^ from cetyltrimethylammonium chloride (CTAC) can serve as a more compact capping agent for the Ag{100} facets. The shorter “tail” of CTAC also helps prevent bulky steric interactions at corners and edges, allowing for the production of sharper cubes.

## 3 Toward Robust and Rational Synthesis of Ag Nanocubes

### 3.1 Optimization of Reagents

**Figure 4:**
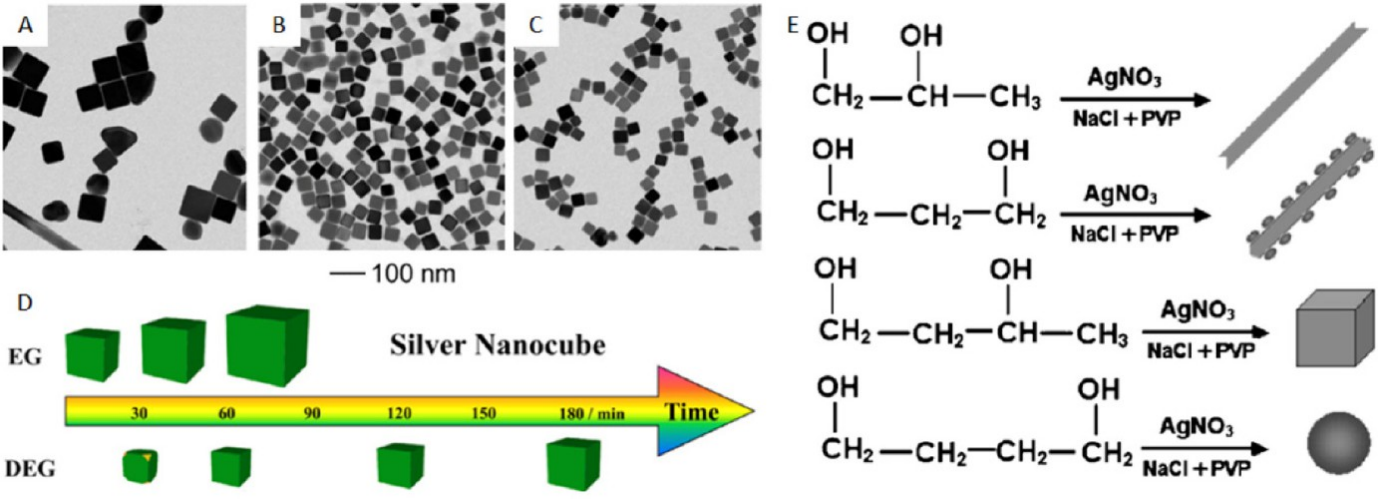
Optimization of reagents for the polyol synthesis of Ag nanocubes. (A–C) TEM images of Ag nanoparticles synthesized under varying concentrations of NaHS and HCl. (A) Low NaHS and high HCl concentrations. (B) Intermediate NaHS and HCl concentrations. (C) High NaHS and low HCl concentrations. (D) Schematic showing the difference in temporal size development of Ag nanocubes in EG vs DEG. (E) Illustration of the various products obtained from the corresponding polyols. (A–C) Reproduced with permission from ref (55). Copyright 2010 John Wiley & Sons. (D) Reproduced with permission from ref (44). Copyright 2013 American Chemical Society. (E) Reproduced with permission from ref (59). Copyright 2008 The Chemical Society of Japan.

A typical polyol synthesis of Ag nanocubes involves the use of a Ag(I) precursor, a reducing agent, a capping agent, and various ionic species. Silver salts are generally known to be sparingly soluble in most solvents, with the notable exception of AgNO_3_. This has made AgNO_3_ the default choice for many synthetic protocols developed in the early days, including those for Ag nanocubes. The NO_3_^–^ ions, however, may not simply act as a spectator.^22,41,42^ As discussed in the previous section, they can be combined with protons to promote the formation of HNO_3_, causing etching and dissolution of the Ag nanoparticles. Although this might be a desired process for the generation of single-crystal seeds, the addition of new pathways can muddy mechanistic understanding. In addition, the elevated temperature typically used for a polyol synthesis of Ag nanocubes may unpredictably cause NO_3_^–^ to decompose, contributing to the poor reproducibility of a synthesis.^55^ Our follow-up studies identified CF_3_COOAg as a more reliable alternative.^44,55−58^ The elimination of NO_3_^–^ not only led to a more robust synthesis but also offered a clearer understanding of how NaHS and Cl^–^ influence the outcome of a synthesis (Figure 4A–C). Furthermore, excluding the additional etching pathway makes it easier to adjust the reduction kinetics so that particle size can be easily controlled by monitoring changes to the UV–vis spectrum.

While most reports assume that Ag is being introduced into the synthesis as Ag^+^, this might not always be the case. Either AgCl or AgBr can also be formed *in situ* when a halide source is involved.^56,58,60−64^ In this case, the AgCl or AgBr precipitates out of solution and subsequently becomes a precursor to elemental Ag. It is well documented that both AgCl and AgBr are sensitive to visible light and can readily undergo photoreduction.^8^ For example, when the precursor solution of AgNO_3_ was aged at room temperature under fluorescent lighting in EG for just 5 min before injection into heated EG, the proportion of multiply twinned nanoparticles drastically increased as compared to a freshly prepared solution. In this case, only penta-twinned nanowires would be obtained in the final product.^65^ To regain nanocubes, the concentration of HCl had to be increased by five times relative to the synthesis involving freshly prepared AgNO_3_. Thus, the actual form of Ag(I) precursor involved in a synthesis can have a major impact on the outcome.

Despite the moderate reduction potential, it is difficult to generate elemental Ag from its salt precursor.^66^ As such, similar polyol reduction of metal precursors such as Pd(II) or Pt(II) occur at much lower temperatures.^67,68^ This prompted us to investigate the reducing power of EG at elevated temperatures and identified glycolaldehyde (GA) as the likely actual reducing agent.^69^ When EG was heated to 150 °C, it was oxidized to GA in the presence of oxygen. This process was then turned into a positive feedback loop when Ag(I) was reduced to generate Ag particles whose surface could catalyze the further oxidation of EG to GA. When EG was replaced with DEG, the longer hydrocarbon portion lowered its relative reduction power by diluting the active groups.^44^ As a result, the growth of nanocubes was slowed down and smaller sizes could be reliably obtained at distinct time points (Figure 4D). Similarly, the addition of glycerol increased solution viscosity, slowing down the diffusion of Ag particles and precursor in the solution to effectively decelerate the reaction kinetics.^70^ When the type of polyol was further varied, the same protocol could result in vastly different morphologies.^59^ Interchanging 1,2-propylene, 1,3-propylene glycol, 1,3-butylene glycol, and 1,4-butylene glycol was found to give Ag nanowires, nanorods with clusters, nanocubes, and spheres, respectively (Figure 4E). Altogether, it is clear that changing the position of the OH groups along a hydrocarbon chain was significant enough to affect both the reaction kinetics and product morphology.

The addition of a sulfide species pushes the synthesis of Ag nanocubes from purely a research endeavor to industrial relevance by significantly cutting down the reaction time.^45^ The duration of such a synthesis can also be reduced even more to merely seconds by switching to microwaves as a source to heat the reaction mixture.^71^ However, before large scale syntheses can be reliably achieved, a few obstacles still need to be overcome. First, Na_2_S is extremely hygroscopic, making it difficult to measuring out an exact amount of this solid.^46,72,73^ Three strategies have been explored to remedy this problem. The first is to run reactions in tandem with varying amounts of Na_2_S.^46^ At least one of the samples will produce Ag nanocubes in high purity. However, this does mean that a majority of the samples will be discarded, which, is not ideal for high throughput. The second strategy is to only use freshly purchased Na_2_S and heat the solid before synthesis to ensure it is completely dehydrated.^72^ Here the difficulty lies in the acquisition of fresh reagents. The third strategy is to utilize a continuous flow reactor and manipulate the flow rate of the Na_2_S phase until high-quality nanocubes are produced.^73^ The use of a continuous flow reactor also has the benefit of easy scaling-up as it eliminates the inhomogeneity intrinsically associated with a large reaction vessel.

Another method used for reliably scaling up sulfide-mediated syntheses is the use of Ar protection.^47^ While oxidative etching can be used to eliminate the multiply twinned seeds, the use of Na_2_S or NaHS circumvents this necessity. When a sulfide is present, it immediately reacts with Ag^+^ to generate small, insoluble Ag_2_S clusters, which then act as seeds for further growth. If self-nucleation does not occur, no twinned particles will be created, and oxidative etching will no longer be necessary. Preheating EG in atmosphere allows for the formation of GA to aid in reduction. However, the continued presence of O_2_ will only etch the desirable particles, lowering their quality. Protecting the synthesis by maintaining a flow of Ar gas ensures uniformity for the nanocubes even in large batch syntheses. The as-obtained nanocubes could be further reacted with an aqueous solution of polysulfide (Na_2_S_*x*_) to help preserve the {100} facets on the side faces.^74^ In this process, the corners were selectively sulfurized to transform the Ag nanocubes into an Ag–Ag_2_S hybrid that could better resist corner truncation in solution over time.

Capping agents play a major role in shape-controlled syntheses, and Ag nanocubes are no exception. The first published synthesis used PVP for dual purposes as a capping agent because of its known affinity toward the Ag{100} facets, as well as a colloidal stabilizer.^27^ Since this first protocol was published, essentially all following reports have also used PVP. Generally, a capping agent is believed to work by preferentially binding to a specific type of facet and thus altering the energy landscape of a system.^75^ However, a recent study of the adsorption isotherms of PVP on various types of Ag facets suggested that this explanation might be incomplete.^76^ As expected, PVP did adsorb more strongly on the Ag{100} facets, but a Wulff construction utilizing the calculated equilibrium adsorption constants indicates that this difference alone was not significant enough to induce a cubic morphology. This trend was also claimed to be supported by the observation in which increasing the concentration of PVP would result in octahedral nanocrystals enclosed by {111} facets. This claim was, however, contradictory to the observations reported in other studies,^77,78^ where Ag cubic seeds were found to grow into larger nanocubes in the presence of adequate PVP and {111} facets only stated to appear on the surface when the concentration of PVP in the reaction solution became inadequate. Furthermore, it should be pointed out that the adsorption isotherms of PVP on Ag nanocrystals with different shapes need to be carefully re-evaluated to make sure that the measurements are correct and accurate as the surface of the nanocrystals could be easily precontaminated by other species. In a computational study, PVP was proposed to form a thicker layer on the {100} facets, greatly reducing the rate of atom deposition and thus resulting in a shift in morphology.^79^ Taken together, future studies are still welcome, and the more likely answer is a combination of thermodynamic factors with kinetic ones.

Similarly, both Cl^–^ and Br^–^ could function in a number of ways within the same synthesis. Because both are halides, Cl^–^ and Br^–^ in large part behave the same way. Their small size allows for better capping because there is no steric hindrance.^58^ In contrast, PVP is a much bulkier macromolecule, making it difficult to achieve sharp corners when particle size is reduced. Both Cl^–^ and Br^–^ react with Ag^+^ to precipitate out as AgCl and AgBr, respectively.^56,58,60−64^ When manipulated correctly, this technique can create a fast burst of nucleation that favors the formation of single-crystal seeds, followed by slow reduction to allow for an easy control over the size.^58,63,64^ While Cl^–^ is more commonly used to promote oxidative etching because of its presence as a contaminant in the original polyol synthesis, Br^–^ can serve the same role.^35,58^ Likewise, both ions have the same tendency to promote anisotropic growth for the production of nanobars when the concentration of Ag^+^ becomes sufficiently low.^58,63,80^

Briefly touched on above is also the importance of reaction atmosphere. Most syntheses rely on oxidative etching, which, requires the participation of O_2_.^35^ Because the O_2_ in these syntheses is simply derived from air, its levels are often not regulated and can result in inconsistencies depending on the laboratory environment. To better control this, a simple fix is to close the reaction vessel.^81^ Closing the reaction vessel remedies the evaporation and condensation cycle of GA and ensures the slow consumption of O_2_ gas while NO is generated to promote nucleation. The concentration of O_2_ can also be controlled by the purposeful injection of this gas into the reaction solution.^82^ Adjusting the flow rate of O_2_ was found to control the etching rate of particles in the solution. This method provides an easy knob to adjust the type of twinning in the final product. In the case involving Na_2_S or NaHS to generate Ag_2_S seeds, O_2_ is no longer necessary,^47^ but the reaction can benefit from the protection of Ar gas to prevent unnecessary etching.

### 3.2 Seed-Mediated Growth

Seed-mediated growth is an effective method to grow Ag nanocubes with different sizes while maintaining shape uniformity. Compared to the one-pot approach, where homogeneous nucleation and growth tend to be entangled, seed-mediated growth allows for separate control to optimize parameters for nucleation and growth events, thereby avoiding issues such as diversity in twin structure and polydispersity in size.^83^ Seed-mediated growth of Ag nanocubes typically requires the use of well-defined single-crystal seeds, a capping agent for the Ag{100} facets, and an optimal reaction temperature. Seeds made of different metals (including, for example, M = Ag, Au, and Pd) have been demonstrated for the generation of M@Ag nanocubes with controllable edge lengths in the range of 13–200 nm.^49−52,77,84^ The edge length can be conveniently adjusted by simply varying the amount of Ag(I) precursor relative to the number of seeds used for growth. In the case of Ag-based seeds, the products have the same monometallic composition while other metals would result in a bimetallic core–shell structure.

**Figure 5:**
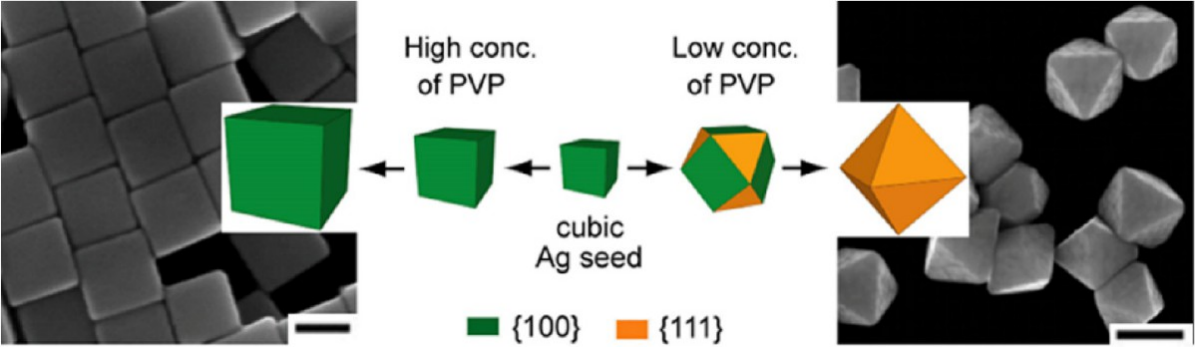
Schematics and corresponding TEM images of the products growing from Ag cubic seeds at different PVP concentrations. The scale bars correspond to 100 and 200 nm for the cubes and octahedra, respectively. Reproduced with permission from ref (78). Copyright 2012 American Chemical Society.

Capping agents play a vital role in determining the final crystallographic facets exposed on the nanocrystals. To this end, we conducted a quantitative analysis of the role played by PVP in the seed-mediated growth of Ag nanocrystals from Ag cubic seeds (Figure 5).^78^ The final nanocrystals adopted different shapes depending on the initial concentration of PVP. If the initial concentration was above a critical value of 1 mM, the 40 nm cubic seeds grew into larger nanocrystals while maintaining the cubic shape. In contrast, if the initial concentration was below a critical value of 0.1 mM, the seeds would evolve into cuboctahedra enclosed by a mix of {100} and {111} facets, and eventually octahedra covered by {111} facets.

**Figure 6:**
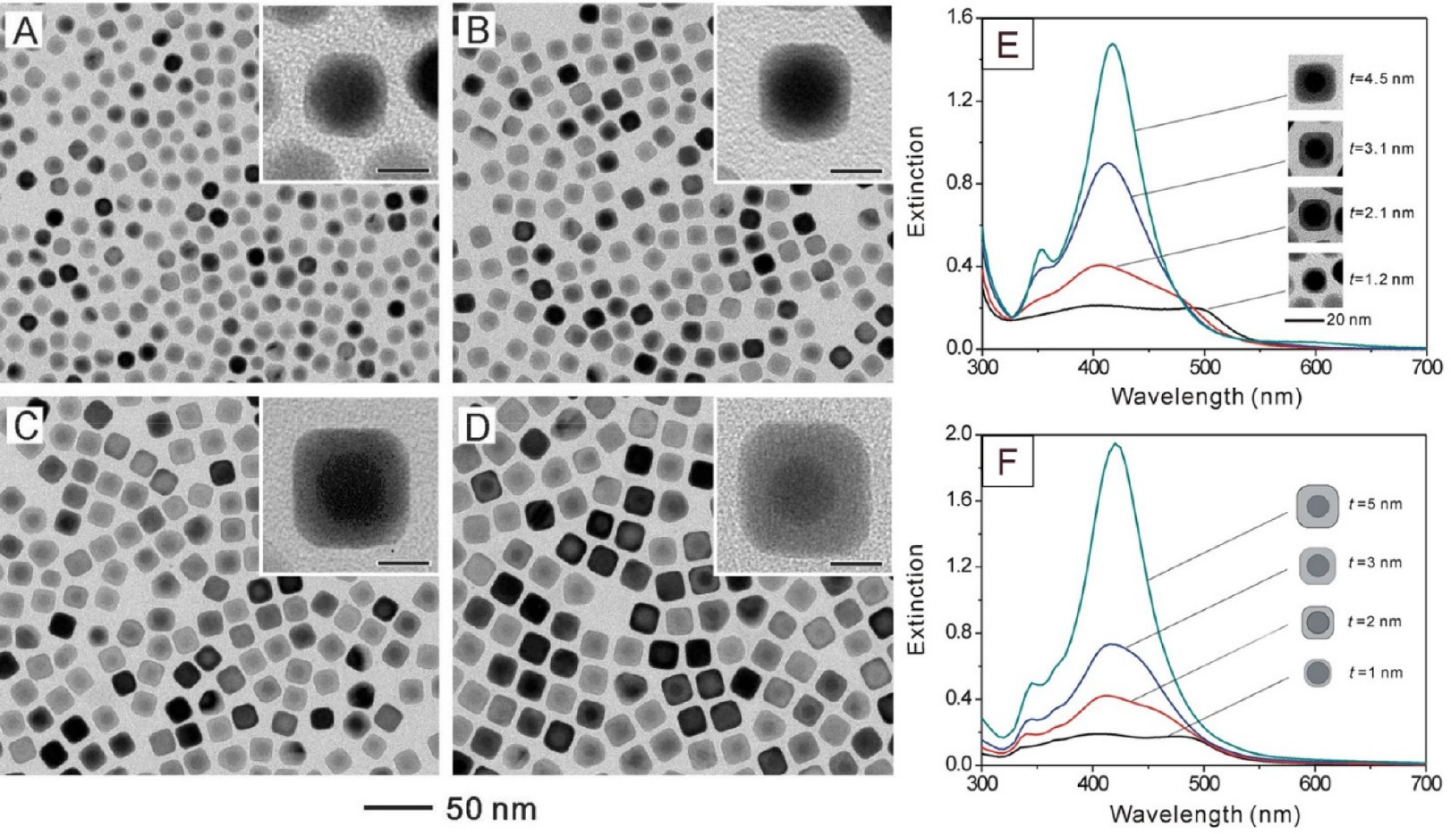
Seed-mediated growth of Au@Ag nanocubes. (A–D) TEM images of Au@Ag core–shell nanocubes obtained when using different volumes of AgNO_3_ at a fixed number of Au seeds. Insets show TEM images at higher magnifications, with scale bars corresponding to 8 nm. (E, F) Experimental and calculated UV–vis spectra of the Au@Ag nanocubes featuring Ag shells of different thicknesses. Modified with permission from ref (50). Copyright 2010 American Chemical Society.

Other than PVP, Cl^–^ is another commonly used capping agent toward Ag{100} facets, especially in an aqueous system. To this end, Au nanospheres have been used as seeds to grow Au@Ag core–shell nanocubes with edge lengths of 13.4–50 nm with the assistance of CTAC.^50^ In the presence of ascorbic acid (AA) and gentle heating at 60 °C, the AgNO_3_ precursor was readily reduced to Ag on the surface of the single-crystal Au spherical seeds for the generation of Au@Ag concentric nanocubes due to the strong capping of Cl^–^ dissociated from CTAC toward Ag{100} facets. By varying the ratio of AgNO_3_ to Au seeds, the thickness of the Ag shells could be readily tuned from 1.2 to 20 nm (Figure 6A–D).

The uniformity in shape and the ability to fine-tune the thickness of the Ag shell in a seed-mediated synthesis allow one to precisely tailor the LSPR properties of the Au@Ag core–shell nanocubes. Figure 6E shows UV–vis spectra of the Au@Ag core–shell cubes with Ag shells of different thicknesses. At a thin shell thickness of 1.2 nm, the Au@Ag nanocubes showed two LSPR peaks located at *ca*. 510 and 410 nm, corresponding to the contributions from the Au core and the Ag shell, respectively. As the thickness of the Ag shell increased, the peak intensity of the Au core decreased and only one single peak corresponding to the Ag component remained when the thickness was increased to 3.1 nm and beyond. This result was consistent with the trend observed in theoretical calculations where the discrete dipole approximation (DDA) method was used to calculate the extinction spectra of Au@Ag core–shell nanocubes. The results presented in Figure 6F also suggested that the incident light could only penetrate Ag shells with a threshold thickness around 3 nm. The precise control over size and sample uniformity enabled by seed-mediated growth has great potentials in obtaining Ag nanocubes with desired optical and catalytic properties.

### 3.3 From Polyol to Organic and Aqueous Systems

Polyol synthesis has so far been the most widely used and successful route of synthesis to obtain Ag nanocubes. These syntheses have offered samples in high purity while their edge length can be controlled in the range of 13–400 nm. Preparation of metal nanocrystals in liquid media other than polyols, on the other hand, could potentially offer advantages such as economic production, flexibility in surface functionalization with organic ligands, as well as abilities to catalyze organic reactions and assist interfacial self-assembly.^57^ However, switching from polyols to other solvents is not straightforward and often involves multifaceted challenges. For example, multiply twinned nanoparticles are usually the dominant products due to the poor control over homogeneous nucleation, and most of the conventionally used precursors are inorganic salts that might face insolubility issues in nonpolar solvents.

**Figure 7:**
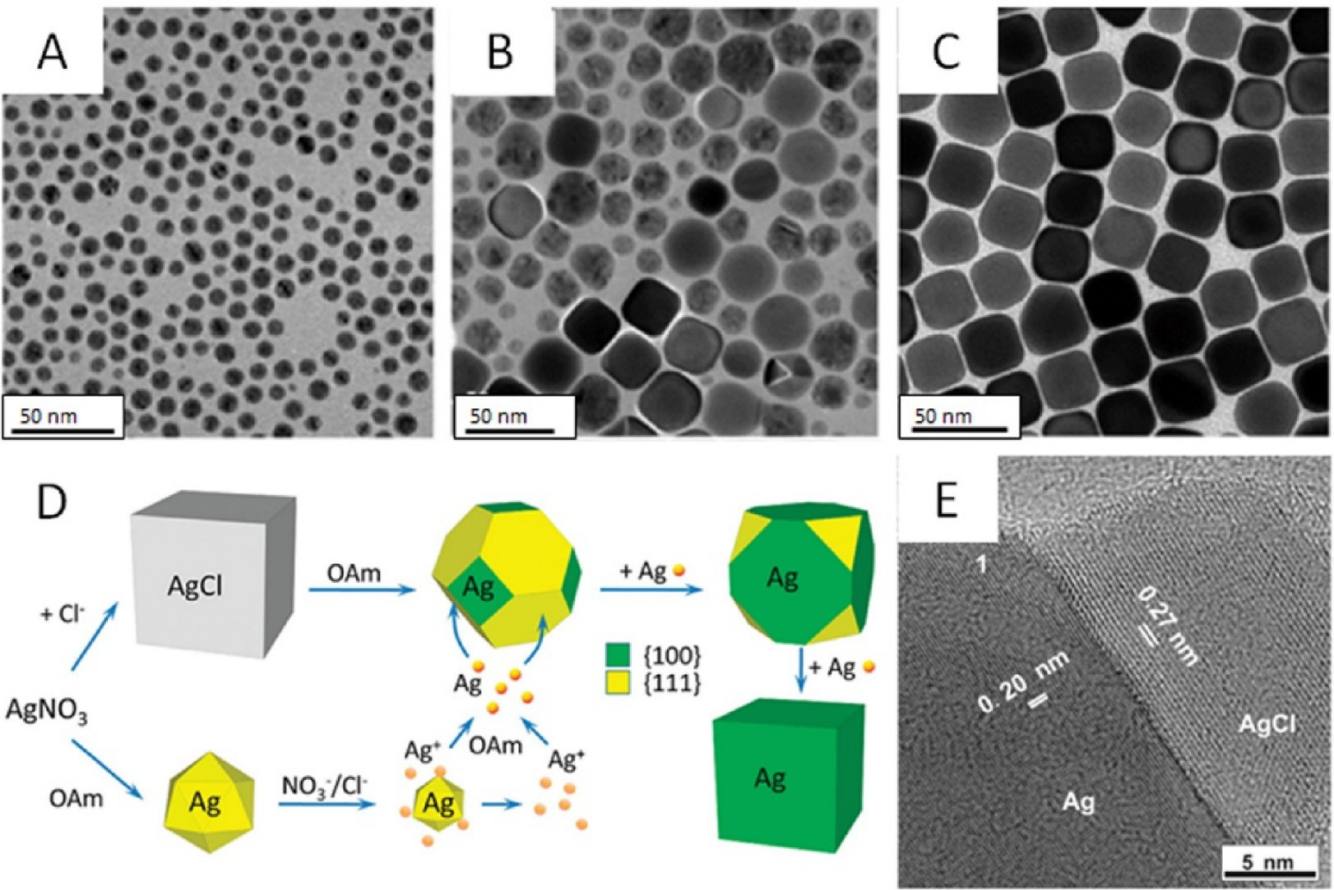
(A–C) TEM images of Ag nanocubes taken at (A) 1, (B) 4, and (C) 48 h into a synthesis conducted in DCB. (D) Schematic showing the evolution of single-crystal seeds into Ag nanocubes in a binary mixture of solvents. (E) TEM image of a Ag nanocube grown in an aqueous solution mediated by the formation of AgCl. (A–C) Reproduced with permission from ref (57). Copyright 2013 Royal Society of Chemistry. (D) Reproduced with permission from ref (62). Copyright 2010 American Chemical Society. (E) Reproduced with permission from ref (56). Copyright 2016 American Chemical Society.

Nevertheless, researchers have developed strategies, including introduction of oxidative environments and special organometallic complexes, for conducting the synthesis in organic media other than polyols. In one study, Liz-Marzán and co-workers demonstrated the synthesis of single-crystal Ag nanocubes in 1,2-dichlorobenzene (DCB) by employing oleylamine as both a reductant and a capping agent, with no need for additional oxidative etchant (Figure 7A–C).^57^ The formation of Ag nanocubes involves the reduction of AgNO_3_ by oleylamine (OAm) at an elevated temperature in DCB. After 1 h into the reaction, Ag spherical nanoparticles with sizes of 8–10 nm were formed (Figure 7A). High-resolution TEM images reveal a multiply twinned structure for these nanoparticles. When the reaction progressed to *t* = 5 h, a small population of single-crystal, polyhedral Ag nanocrystals coexisted with the twinned particles (Figure 7B). Afterward, the twinned particles kept decreasing in proportion while the single-crystal particles gradually evolved into Ag nanocubes with an average edge length of 26 nm as the final product at *t* = 48 h (Figure 7C). The conversion from multiply twinned to single-crystal structures were attributed to the presence of NO_3_^–^ and Cl^–^ ions from the precursor and the solvent, respectively, which could work with the dissolved O_2_ to induce slow oxidative etching and thereby dissolution of defect sites inside the multiply twinned Ag particles at the relatively high temperature and prolonged reaction time. Our group also reported a similar synthesis of Ag nanocubes in a hydrophobic solvent by introducing the Fe(III) species in the form of FeCl_3_ or Fe(acac)_3_ into isoamyl ether as an effective etchant to produce single-crystal Ag nanocubes with an edge length as small as 13.5 nm.^85^

As demonstrated by Sun and co-workers, the hydrophobic synthesis of Ag nanocubes can also be extended from a single solvent to a binary mixture of organic solvents.^62^ A mixture of octyl ether and OAm was used to dissolve dimethyldistearylammonium chloride (DDAC) at 260 °C, to which an OAm solution of AgNO_3_ was rapidly injected. The reaction solution immediately turned milky yellowish and both AgCl and Ag nanoparticles were identified as the initial products at the early stage of the synthesis (Figure 7D). The AgCl nanoparticles were formed through the precipitation reaction between Ag^+^ and Cl^–^ ions, and the Ag nanoparticles were generated through the reduction of AgNO_3_ by OAm. The AgCl nanoparticles were then quickly reduced to Ag by OAm at an elevated temperature, which resulted in a rapid disappearance of the milky yellow color, followed by a deep yellow color again after 1 min into the synthesis. The single-crystal particles gradually increased in size and evolved into cubes accompanied by the decrease in size and population of the multiply twinned particles. The final products contained uniform Ag nanocubes in high purity, together with an average edge length of 34 nm.

Despite the success of syntheses in both polyol and hydrophobic organic solvents, these kinds of syntheses typically require a high reaction temperature and an enhanced oxidative environment. These conditions inevitably induce slight corner and edge truncation for the Ag nanocubes. Polyol synthesis is also sensitive to impurities (such as trace amounts of Cl^–^ and Fe(II)/Fe(III) ions associated with the manufacturing and/or storage process), water, and O_2_ content, posing challenges for reproducing the synthesis. In addition, the use of organic solvents and relatively elevated temperatures raises environmental and economic concerns for these protocols. A water-based system can potentially address these issues. The first aqueous synthesis of Ag nanocubes was published in 2004 by Yam and Yu, just two years after the report of the polyol synthesis.^60^ The aqueous method was based on the “silver mirror reaction” modified with the addition of hexadecyltrimethylammonium bromide (CTAB). The synthesis was performed under hydrothermal conditions and was proposed to involve the following reactions:
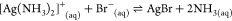
1
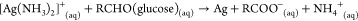
2The presence of CTAB led to the precipitation of AgBr, which was then slowly dissolved throughout the synthesis as the equilibrium was shifted with the reduction of AgBr into Ag. Although the method was successful, it lacked a mechanistic explanation for the formation of nanocubes. A follow up paper in 2005 explored the effect of CTAB concentration on the final products but did not offer much further explanation of the mechanism.^61^ Because this protocol involved the formation of Ag(NH_3_)_2_OH, an explosive and hazardous compound, it is not hard to understand why it did not see any widespread adoption.

In 2016, we reported an aqueous method involving the use of CTAC to generate Ag nanocubes with average edge lengths tunable in the range of 35–95 nm.^56^ The reaction started with the formation of AgCl microscale octahedra upon mixing CF_3_COOAg with CTAC. In the presence of room light and a proper reducing agent such as AA, Ag_n_ nuclei were generated on the surface of the AgCl octahedra at 60 °C, followed by their evolution into single-crystal seeds and eventually Ag nanocubes (Figure 6E). The use of Cl^–^ as a selective capping agent toward the Ag{100} facets and the relatively low reaction temperature enabled the formation of Ag nanocubes with sharp corners and edges. The addition of a trace amount of FeCl_3_ ensured that the twinned nanoparticles were etched away, only leaving behind single-crystal seeds to grow into nanocubes.

A comparative summary of all the synthetic strategies discussed in this review can be found in Table 1.

**Table 1:** Summary of All Synthetic Strategies Described, along with Their Benefits and Drawbacks

Synthesis	Pros	Cons	Refs
Original Polyol Synthesis	-First synthesis of Ag nanocubes	-Poor reproducibility	(27)
		-Limited size control	
Oxidative etching (with Cl^–^/O_2_)	-Good reproducibility	-Long reaction time	(35)
	-Good uniformity	-Creating twinned seeds as an intermediate	
Oxidative etching (with Fe(III)/Fe(II))	-Faster reaction time than the protocol involving Cl^–^/O_2_	-Highly dependent on concentration and temperature	(36, 56, 85)
		-Creating twinned seeds as an intermediate	
Addition of proton/formation of NO_3_^–^	-Increased uniformity	-Slowed reaction rate	(22, 41, 42)
		-Creating twinned seeds as an intermediate	
Sulfide-mediated	-Fastest reaction time	-Sensitive to O_2_	(44−47, 55, 58, 74, 77)
	-Creating only single-crystal seeds and products		
Seed-mediated	-Precise size control	-Multiple steps	(49−52, 63, 77, 80, 84)
	-Possible introduction of second metal		
CTAB/CTAC	-Less truncated corners	-Capable of inducing changes to aspect ratio	(50, 56, 63, 80)
	-Smaller particles		
CF_3_COOAg	-Simplified reaction pathway	-Elimination of some etching pathways	(44, 55, 56, 58, 63, 77, 85)
	-Avoiding species from NO_3_^–^ decomposition		
Aqueous	-Sharp corners	-Sensitive to light	(56)
	-Mild reaction conditions	-Indirect formation of nanocubes through AgCl	
	-Green chemistry		
Polyol (not EG)	-Smaller nanocubes through kinetic control	-Leading to completely different morphologies	(44, 59)
Organic Solvent	-Increased uniformity	-Limited reagent solubility	(57, 62, 85)
	-Small nanocubes	-High temperatures	
		-Highly oxidative environment, leading to truncation	
Hydrothermal	-Sharp edges	-Hazardous precursor	(60, 61)
	-Good uniformity	-Unclear mechanism	
PVP	-Low toxicity	-Truncated corners	(27, 35, 36, 78)
	-Easy to work with	-Larger particles	
		-Final shape sensitive to concentration	

## 4 Applications

Silver is probably the most valuable material in plasmonics as it offers many advantages over Au, Cu, Al, and Li and some other metals known to support surface plasmons in the visible and near-infrared regions. Specifically, Ag is able to support a strong surface plasmon across the spectrum from 300–1200 nm.^86^ Further, Ag has a long history of being used as a catalyst, particularly in the plastics industry.^10^ The combination of these properties, alongside precise shape control, has led to a plethora of applications in many different areas.^87,88^ The relatively low reduction potential of Ag also opens the opportunity for easy incorporation of other metals through galvanic replacement.^66,89^ When combined with Au, Pd, Pt, Rh, and other metals, the plasmonic and catalytic capabilities of Ag can be further enhanced.^89^

### 4.1 LSPR

**Figure 8:**
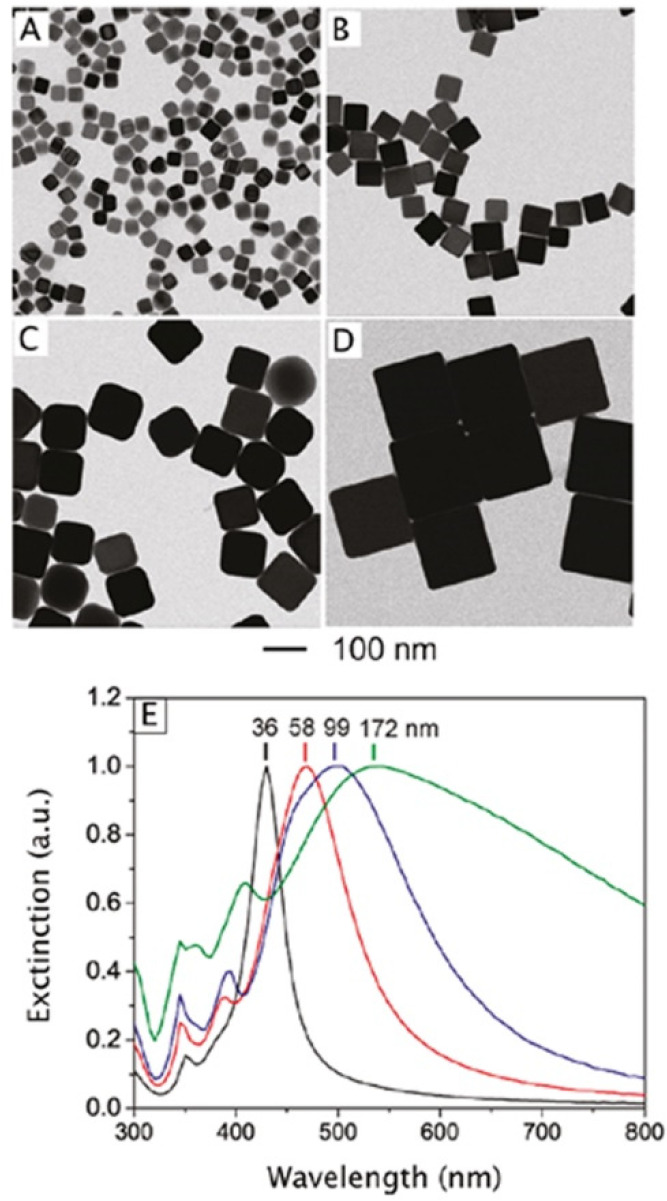
(A–D) TEM images of Ag nanocubes with edge lengths of 36, 58, 99, and 172 nm, respectively. (E) UV–vis spectra recorded from aqueous suspensions of Ag nanocubes with different edge lengths, indicating that the main LSPR peak was red-shifted as the edge length was increased. Reproduced with permission from ref (77). Copyright 2010 American Chemical Society.

This optical property arises from the collective oscillation of conduction electrons in a nanocrystal.^90^ By controlling the size and/or shape of Ag nanocrystals, one can tailor their LSPR features to suit a range of applications, including optical sensing, SERS, and near-field optical microscopy.^20^ Our early work focused on the synthesis of Ag nanocrystals with different shapes because this parameter offers a more sensitive knob than size to tune the LSPR characteristics.^19^ Nanocubes are particularly interesting because both their measured and their calculated UV–vis spectra exhibit more LSPR peaks than nanospheres. This is due to the larger number of distinct directions to polarize the electrons, as enabled by the less symmetric shape. In addition, Ag nanocubes displayed a more intense dipole peak that was red-shifted relative to that of the spherical counterpart. This feature is commonly observed for nanocrystals bearing sharp corners,^19^ which can reduce the restoring force for electron oscillation and thereby cause a red-shift to the resonance peak. In a later study, we also examined the size dependence of the LSPR properties of Ag nanocubes (Figure 8).^77^ Increasing the edge length of the nanocubes caused the LSPR peak to continuously red-shift, yielding a more or less linear relationship. Based on this linear correlation, the size of the Ag nanocubes could be precisely controlled by quenching the synthesis when the desired LSPR peak position was reached.

**Figure 9:**
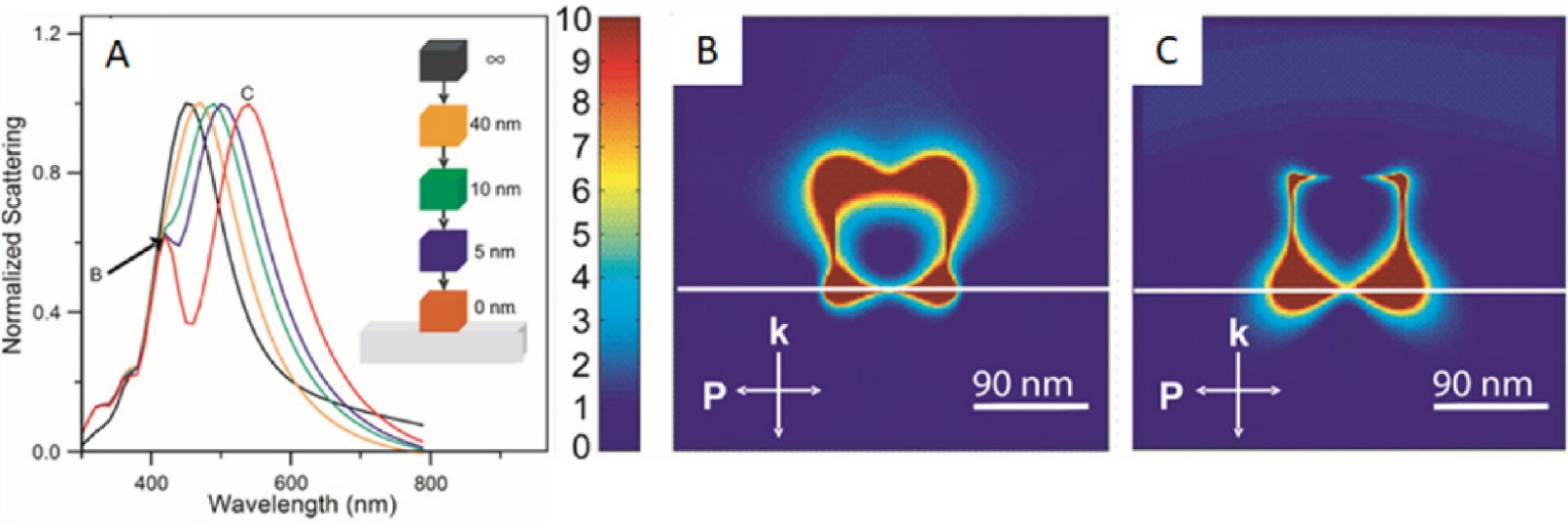
Effect of a glass substrate on the LSPR property of an approaching Ag nanocube. (A) Calculated scattering spectra of a Ag nanocube with an edge length of 90 nm as it approaches a glass substrate. The single peak associated with the dipolar LSPR (black line) begins to split as it approaches and touches the substrate (red line). The peaks resulting from the split (the red trace) are labeled corresponding to the FDTD simulations where (B) shows the plasmon modes away from the substrate and (C) shows the modes adjacent to the substrate. The white line in the simulation represents the glass substrate. Reproduced with permission from ref (20). Copyright 2005 American Chemical Society.

It is often required to deposit the nanocrystals on a solid substrate when performing single-particle measurements in order to fix the location and orientation of the nanocrystals with respect to the environment. To this end, the supporting substrate may have a major impact on the LSPR properties and near-field distribution of the nanocrystals. For instance, it was reported that the major LSPR peak split into two peaks, with one (peak 1, at 430 nm) blue-shifted and the other (peak 2, at 550 nm) red-shifted from the original LSPR peak measured in solution when the Ag nanocube approaches a glass substrate.^20^ According to finite difference time domain (FDTD) calculations, peak 1 could be assigned to the near-field LSPR mode facing away from the glass substrate while peak 2 arose from the near-field LSPR mode toward the substrate (Figure 9).^20^ Large polarizations are induced on both the side near and away from the glass substrate, resulting in the production of two types of resonances. Significantly, the supported Ag nanocubes could serve as a better chemical sensor because the resonance at 430 nm was found to be exceptionally sensitive to changes in its dielectric environment. Altogether, these results clearly demonstrate the size- and shape-dependences of the LSPR properties of a nanocrystal, in addition to the effect of a dielectric substrate.

### 4.2 SERS

Due to their favorable plasmonic properties, Ag nanocrystals exhibit strong SERS enhancement in a wide range of wavelengths from 300–1200 nm, creating “hot spots” (regions with the highest E-field enhancement) to enable highly sensitive detection.^90,91^ In general, SERS enhancements are dependent on the physical parameters of Ag nanocrystals, including size and shape. In an early study, we compared the SERS activities of sharp and truncated Ag nanocubes with sizes in the range of 60–90 nm using 514 and 785 nm lasers.^92^ As the particle size was increased, the SERS activity was accordingly enhanced. Additionally, particles with sharper corners showed more intense SERS signals relative to their truncated counterparts. This trend in SERS enhancement can be largely attributed to the difference in overlap between the laser source and the LSPR as a function of size and degree of truncation.

**Figure 10:**
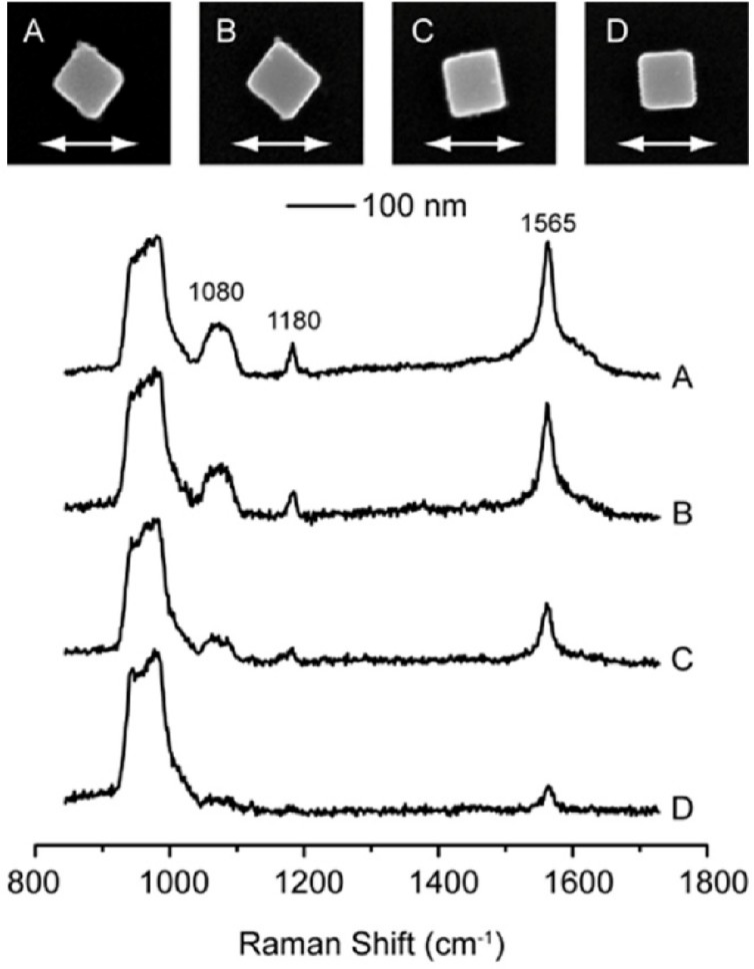
SEM images of individual Ag nanocubes (labeled A–D) that had been deposited on a registered substrate and then functionalized with 1,4-benzenedithiol (1,4-BDT). The 100 nm scale bar applies to all images. The white arrows in the images denote the polarization direction of the incident laser. The corresponding SERS spectra from these particles are stacked below the SEM images. Reproduced with permission from ref (22). Copyright 2007 American Chemical Society.

When a Ag nanocube was deposited on a silicon substrate, the SERS activity of the cube was found to be dependent on its orientation relative to laser polarization.^22^ Specifically, Ag nanocubes with sharp corners were the most active when the diagonal axis was aligned parallel to the laser polarization (Figure 10). The SERS activity was significantly lowered when the cube was oriented with an edge parallel to the laser polarization. Calculations indicate that the difference in SERS activity could be attributed to the variation of the near-field distribution as the orientation of the Ag nanocube was altered relative to laser polarization. It is worth noting that the degree of truncation of the cube could have a crucial impact on the correlation between the SERS activity and the orientation. In general, the truncated Ag nanocube was much less sensitive to orientation than its counterpart with sharp corners.

Subsequent literature reported that hot spots featuring strong and reproducible SERS enhancement could be created by simply depositing individual Ag nanocubes on a Au or Ag substrate.^91^ For Ag nanocubes, the enhancement factor (EF) significantly increased by a factor of *ca*. 250 when a glass substrate was replaced with either a Au or Ag substrate, suggesting the formation of “hot spots” between the nanocube and substrate. For Ag nanospheres, however, the EF was only increased by a factor of 120 when the substrate was switched from glass to Au or Ag. These results indicate that both the electrical property of the substrate and the shape of the particles should be carefully chosen for the creation of “hot spots”. In the case of Ag nanocubes, it is likely that the “hot spots” are formed at the corner sites in proximity to the metal (Au or Ag) substrate, which was validated by plasma etching that removed the probe molecules on the particle’s surface except for the nanocube-substrate interface. When SERS spectra were taken from the same Ag nanocube before and after plasma etching, the strong remaining SERS intensities confirm that the “hot spots” were positioned at the corner sites on the nanocube-substrate interface.

### 4.3 Metamaterials

**Figure 11:**
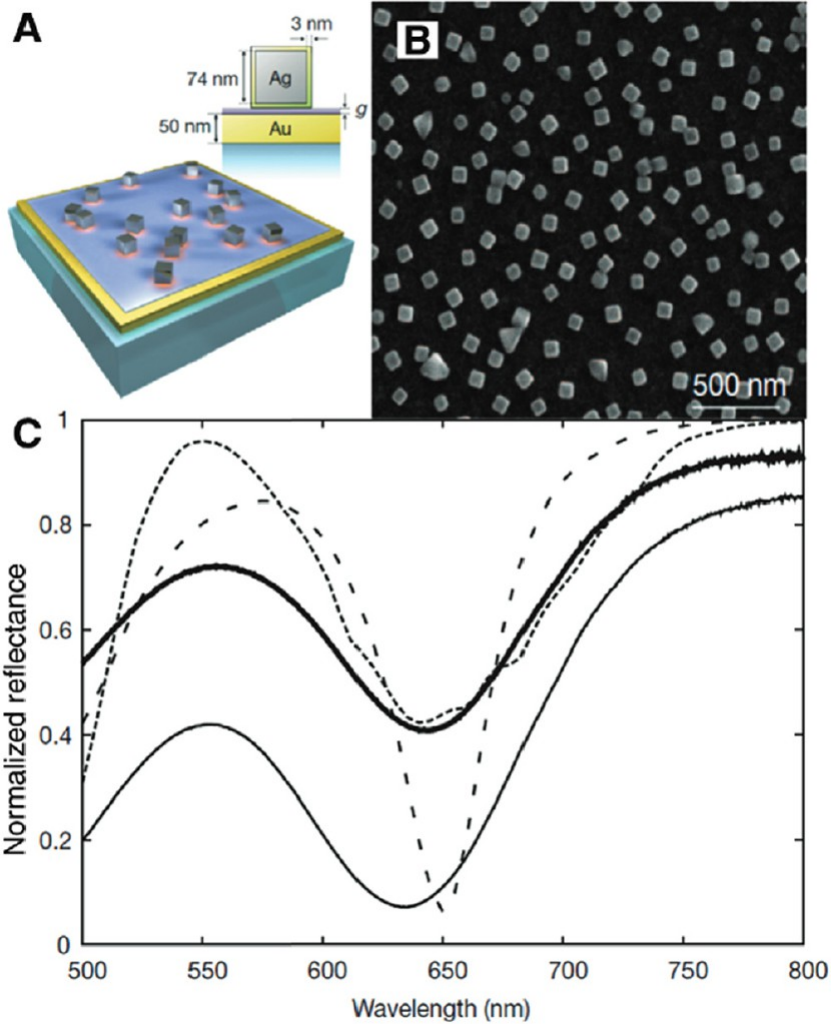
(A) Metamaterial absorbers with tunable reflectance can be fabricated by covering the Au film with Ag nanocubes separated by a 10 nm polymer spacer layer (*n* = 1.54). (B) Top-view SEM image of the as-fabricated optical metamaterial absorber. (C) Experimental reflectance for normal incidence, benchmarked against the Au film, for surface coverages of 7.3% (thin solid line) and 17.1% (thick solid line), compared with simulations of uniform nanocubes (4.2% surface coverage, dotted line) and a model including size dispersion. Reproduced with permission from ref (87). Copyright 2012 Springer Nature.

Both Ag and Au nanocrystals can be assembled into metamaterials to manipulate the absorption/transmission/reflection of sound or electromagnetic waves.^93^ For example, when a monolayer of millions of Ag nanocubes is deposited on a polymer layer supported on a Au film, one would obtain an efficient and cost-effective light absorber for applications related to sensing and energy harvesting (Figure 11).^87,94^ The polymer layer with a controlled thickness can be fabricated through layer-by-layer deposition of poly(allylamine hydrochloride) and polystyrenesulfonate on a Au film. The surface of the polymer layer is then briefly exposed to a colloidal suspension of Ag nanocubes, which randomly adsorb and immobilize on the surface. In an ideal light absorber, both transmittance and reflectance must be minimized. While any opaque material (e.g., the Au film in Figure 11) can be used to eliminate transmittance, it is more difficult to remove reflectance. In the metamaterial, the dielectric polymer spacer separating the Ag nanocubes from the Au film as well as the nanocube diameter play key roles. Choosing the correct polymer thickness and nanocube diameter ensures that the electric and fictitious magnetic currents that occur upon excitation by incident light, are out of phase with each other so that their sum cancels out and no reflectance occurs.

Light absorbers are traditionally fabricated using lithographically patterned metallic structures,^95^ which are expensive and challenging to scale up to the large surface areas required by many applications. In contrast, bottom-up colloidal methods similar to the one described above can be cheap and easily scalable. Since the arrangement of Ag nanocubes does not matter for an isotropic absorber, this method does not rely on any patterning technique. The coverage of Ag nanocubes on the polymer film only needs to be high enough to induce a magnetic current that can offset the electric current from incident radiation. Theoretically, approximately 3% of the surface covered by 70 nm Ag nanocubes can reach almost complete absorption over a narrow and tunable wavelength region. In practice, the size variations among the chemically synthesized Ag nanocubes contributes to a broader and shallower absorption dip in the reflectance (Figure 11). Increasing the coverage of Ag nanocubes up to 17.1% could compensate for the variation in particle size to achieve low reflectance. The size dispersion of Ag nanocubes still needs to be narrowed to obtain a narrow absorption band. This is achievable by improving either synthetic methods or nanoparticle separation techniques. It is also worth mentioning that the film-coupled Ag nanocubes can support both extreme sensitivity of the resonant mode and provide resonance with a character completely different from the classical resonance of film-coupled spherical nanoparticles. Altogether, large-area metamaterials with controlled reflectance are primed for a growing number of applications, including fluorescence spectroscopy, energy-harvesting, and biosensing.^96^

### 4.4 Self-Assembly

**Figure 12:**
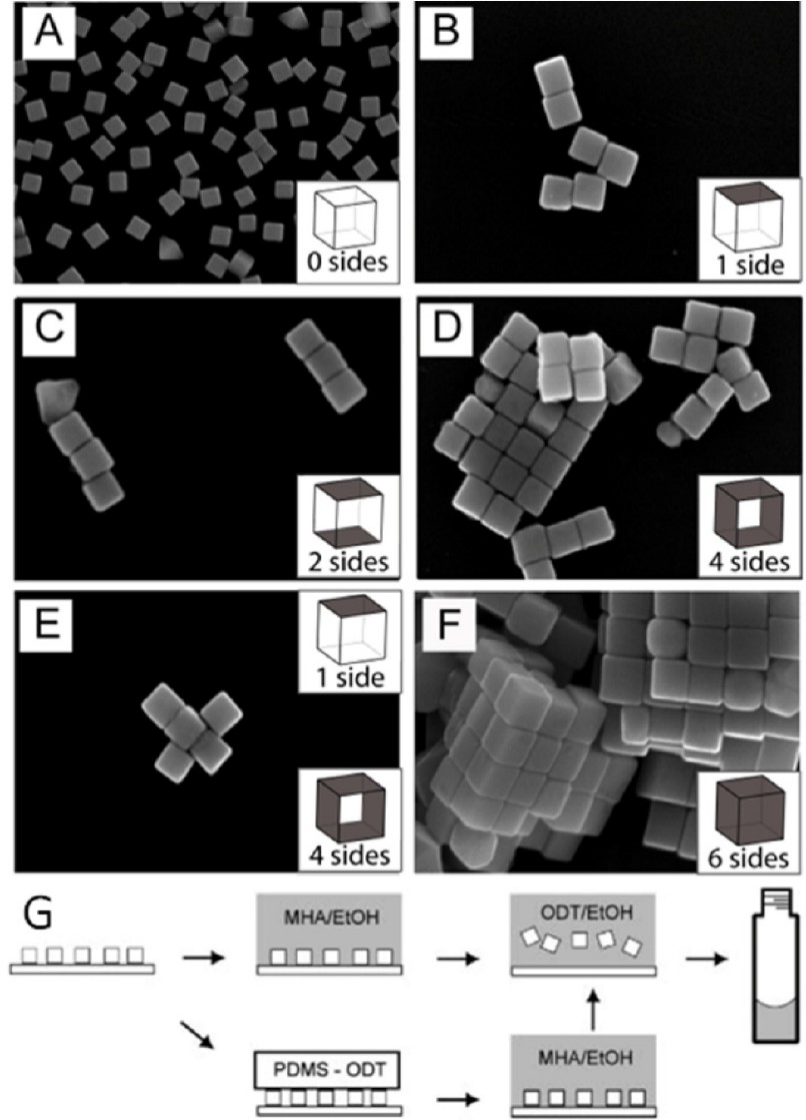
(A–F) SEM images of Ag nanocubes and their assemblies. Unfunctionalized cubes deposited on Si from water are shown in (A) for comparison. Nanocubes whose faces have been selectively functionalized with hydrophilic and hydrophobic thiolate SAMs and then allowed to assemble in water are shown in (B)–(F). The number of faces on each cube that were rendered hydrophobic is indicated in the inset of each panel; the remaining faces on the cube were rendered hydrophilic. In (E), cubes with 4 hydrophobic sides were mixed with cubes that only had one hydrophobic face at a ratio of 1:4 and then allowed to assemble in water. All cubes used in this study had a mean edge length of about 97 nm. (G) Schematic illustrating how the faces of a Ag nanocube are selectively functionalized. Reproduced with permission from ref (100). Copyright 2008 John Wiley & Sons.

The synthesis of noble-metal nanocrystals with well-controlled sizes and shapes has also enabled their use as building blocks for self-assembly.^97^ Going beyond the single particle level, self-assembly offers an effective route to complex nanostructures with new or enhanced optical properties.^97,98^ In a typical self-assembly process, building blocks such as molecules and mesoscopic objects are organized into new structures through noncovalent interactions. The resultant structures are determined by the features encoded on the building blocks, including, for example, topology, shape, and surface functionality. One particularly interesting feature of the building blocks that can likewise be passed onto the assembled structures is anisotropy. Over the past years, diverse nanocrystals with anisotropic shapes have been synthesized but few of them have been explored as building blocks for self-assembly likely because assembly of anisotropic nanocrystals faces significant challenges.^99^ For instance, it remains a difficult task to control the orientation of anisotropic nanocrystals relative to their neighbors or underlying substrate, which is important for plasmonically active materials. Various methods have been developed to organize and align anisotropic building blocks, including nondirected methods, directed assembly via surface modification, external field-directed assembly, and templated self-assembly.^100,101^ Among these methods, surface modification significantly expands the versatility of anisotropic building blocks for self-assembly, but often requires challenging chemistry to modulate the surface properties. To this end, we were able to fabricate five distinct self-assembled structures from surface-modified Ag nanocubes.^100^ Specifically, the side faces of Ag nanocubes were selectively functionalized with either hydrophilic or hydrophobic self-assembled monolayers (SAMs). The nanocubes then spontaneously assembled to reduce their surface energy by reducing their hydrophobic surface area exposed to water. As shown in Figure 12, five distinct SAM-modified Ag nanocubes could be fabricated, depending on the number and positions of the hydrophobic faces. Specifically, the selective functionalization of faces relies on the capability to protect them with a clean Si substrate and functionalizing the remaining faces sequentially with solutions of alkanethiols or poly(dimethylsiloxane) (PDMS)pads inked with alkanethiols. As a result, dimers, liner chains, square or rectangular two-dimensional (2D) sheets, and three-dimensional (3D) cubic lattices were obtained for systems involving 1, 2 (opposite), 4 (two opposite faces were left hydrophilic), and 6 hydrophobic faces, respectively, on each Ag nanocube. Specifically, cubes with one hydrophobic face were functionalized by depositing the as-prepared Ag nanocubes on a clean Si wafer. The nanocubes were then submerged in a solution of mercaptohexadecanoic acid (MHA) in ethanol (EtOH) to functionalize the five exposed faces with hydrophilic MHA. Subsequently, the nanocubes were released from the Si wafer and dispersed in a solution of octadecanethiol (ODT) in EtOH to functionalize the remaining face with hydrophobic ODT. When the order of MHA and ODT was reversed, we obtained nanocubes with five hydrophobic faces and one hydrophilic face. For Ag nanocubes with two hydrophobic faces, they could be fabricated in a similar manner with the introduction of an extra step. Before functionalization with MHA, once deposited on the clean Si surface, the nanocubes would first be printed with ODT using a PDMS elastomeric stamp. The now partially functionalized Ag nanocubes could be submerged in the MHA/EtOH solution to functionalize the four remaining exposed faces. Finally, the nanocubes could be detached and the newly exposed face could be functionalized with ODT as previously described. Likewise, nanocubes with four hydrophobic faces and two hydrophilic faces could be obtained by reversing the order of MHA and ODT.

### 4.5 Seeds for Further Growth

Silver nanocubes have been explored as single-crystal seeds for further growth into a greater variety of Ag nanocrystals such as enlarged cubes, octahedra, and cuboctahedra. Due to the absence of lattice mismatch, the deposition of Ag atoms derived from the added precursor is epitaxial.^83^ In an early study, we demonstrated seed-mediated growth of Ag nanocubes with edge lengths controllable in the range of 30–200 nm by leveraging the Ag cubic seeds from a polyol synthesis (Figure 8).^77^ The Ag cubic seeds could be enlarged by reducing AgNO_3_ in the presence of PVP. It is crucial to use AgNO_3_ instead of CF_3_COOAg as the precursor in the growth process. In the case of AgNO_3_, HNO_3_ is formed, helping eliminate homogeneous nucleation of the Ag atoms and thus prevent the formation of nanocrystals with twin defects and/or different sizes. The size of the resultant Ag nanocubes could be tuned by varying the reaction time, amount of AgNO_3_ added, and/or quantity of Ag seeds.

**Figure 13:**
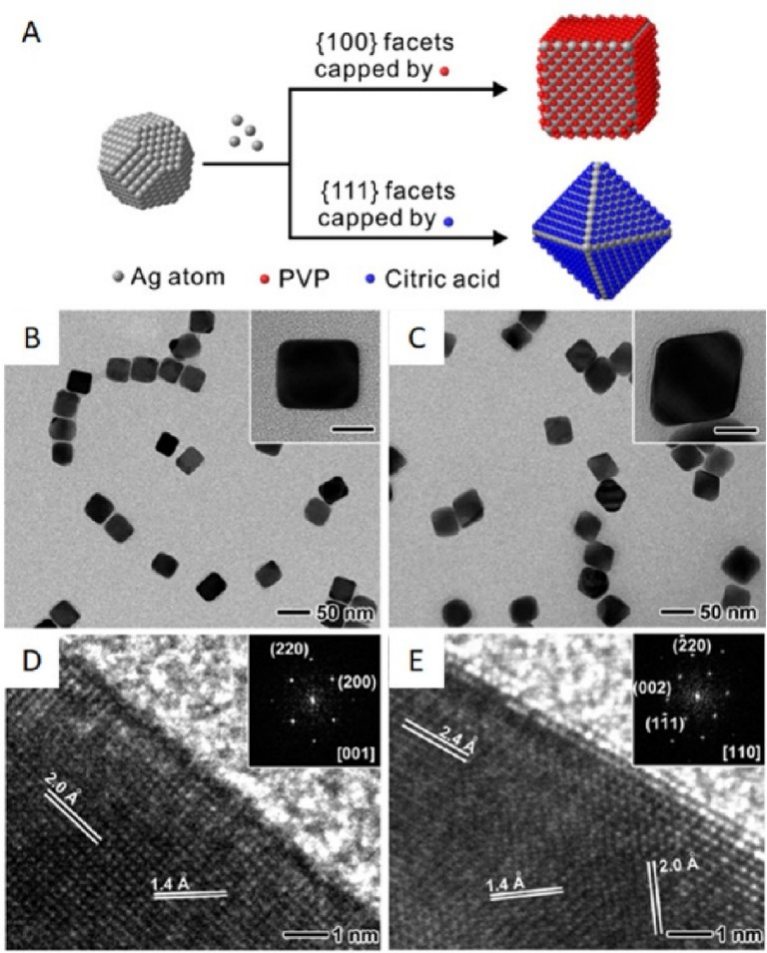
(A) Transformation of single-crystal Ag seeds to nanocubes and octahedra when the growth is carried out in the presence of PVP and citric acid, respectively. (B) TEM and (D) HRTEM images of Ag nanocubes. (C) TEM and (E) HRTEM image of Ag octahedra. Scale bars of the insets in (B) and (C) correspond to 20 nm. The insets in (D) and (E) are electron diffraction patterns. (A) Reproduced with permission from ref (83). Copyright 2017 John Wiley & Sons. (B–E) Reproduced with permission from ref (52). Copyright 2010 American Chemical Society.

Seed-mediated growth also offers an additional method to manipulate the growth rates of different facets and thus achieve shape control. For example, we demonstrated that introducing a caping agent can effectively manipulate growth rates of different facets on Ag nanocrystals (Figure 13).^52^ Consistent with theoretical studies, the addition of citrate led to the formation of Ag octahedra because citrate binds more strongly to Ag{111} than Ag{100} facets. Therefore, growth on the {111} facets was suppressed because of the citrate adsorption while the relatively clean {100} facets gradually disappeared with the deposition of Ag atoms. This eventually leads to the formation of Ag octahedra covered by the citrate-capped {111} facets. In contrast, PVP preferentially binds to the {100} facets, resulting in the formation of enlarged Ag nanocubes or nanobars (as a result of symmetry reduction) enclosed by {100} facets.

**Figure 14:**
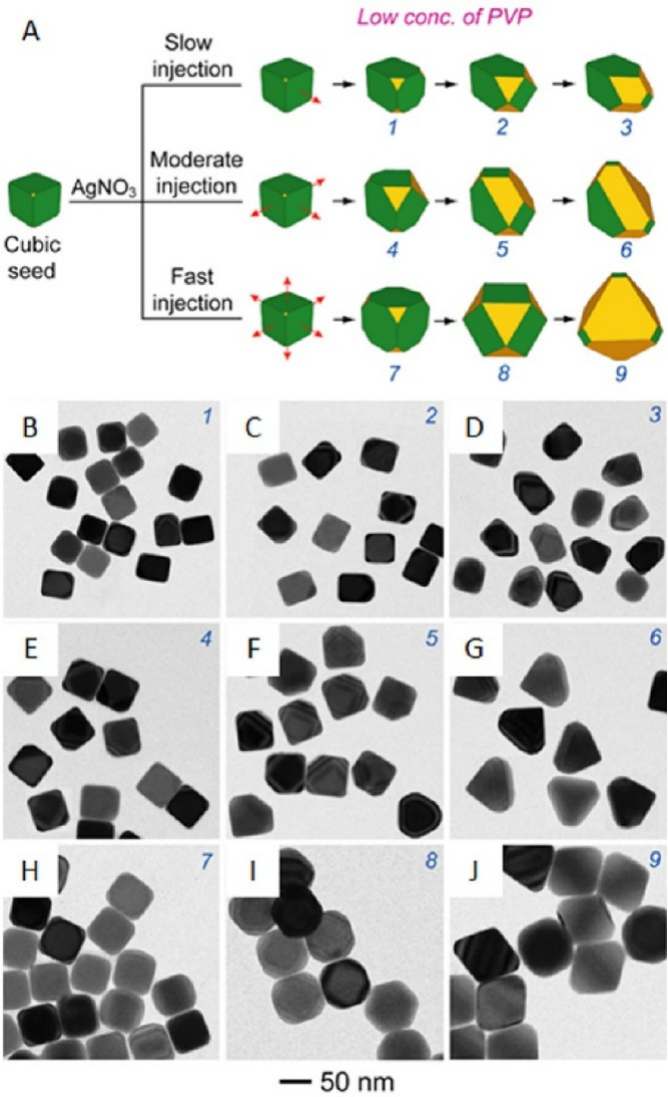
Growth pathways for Ag cubic seeds at a relatively low concentration (1.0 mg/mL) of PVP. (A) Schematic showing the morphological changes for Ag nanocrystals formed under three different injection rates for AgNO_3_ solution. The red arrows indicate the directions of growth. (B–J) TEM images showing the shape evolution of Ag nanocrystals with the growth occurring on (B–D) one, (E–G) three, and (H–J) six faces of a cubic seed. The numbers on 3D models match the numbers on the TEM images. Reproduced with permission from ref (103). Copyright 2012 American Chemical Society.

The aforementioned Ag nanocrystals obtained via seed-mediated growth are highly symmetric in terms of shape, as constrained by the *fcc* lattice of Ag. We also discovered that the cubic symmetry of the seeds could be reduced or broken for the generation of asymmetric nanocrystals.^102,103^ In one study,^102^ aqueous AgNO_3_ solution was added into a mixture of Ag cubic seeds, AA, and PVP with a syringe pump. By controlling the injection rate, the growth of Ag cubic seeds (enclosed by 6 equiv side faces) could be activated for 1, 3, or 6 of the side faces (Figure 14). The slow injection rate of aqueous AgNO_3_ leads to a low flux of Ag atoms, which might only be enough to nucleate from one of the six side faces of a Ag cubic seed, resulting in the formation of a truncated octahedron with five of the six corners removed from the perfect octahedron. When the injection rate was moderate, three adjacent faces of the Ag cubic seed could receive Ag atoms, leading to the formation of a truncated octahedron with three of the six corners removed. At a high injection rate, the concentration of Ag atoms was high enough to provide all the six side faces with Ag atoms, producing a perfect octahedron without corner truncation. A similar strategy was used for Ag overgrowth on Ag cubic seeds, where a limited supply of Ag atoms and strong capping of Cl^–^ ions were employed to cause the evolution of Ag cubic seeds into nanobars with reduced symmetry.^63^ In general, a combination of seed-mediated growth and kinetic control offers new promises for obtaining new types of anisotropic nanocrystals that are challenging to obtain using traditional one-pot synthesis.

### 4.6 Galvanic Replacement

**Figure 15:**
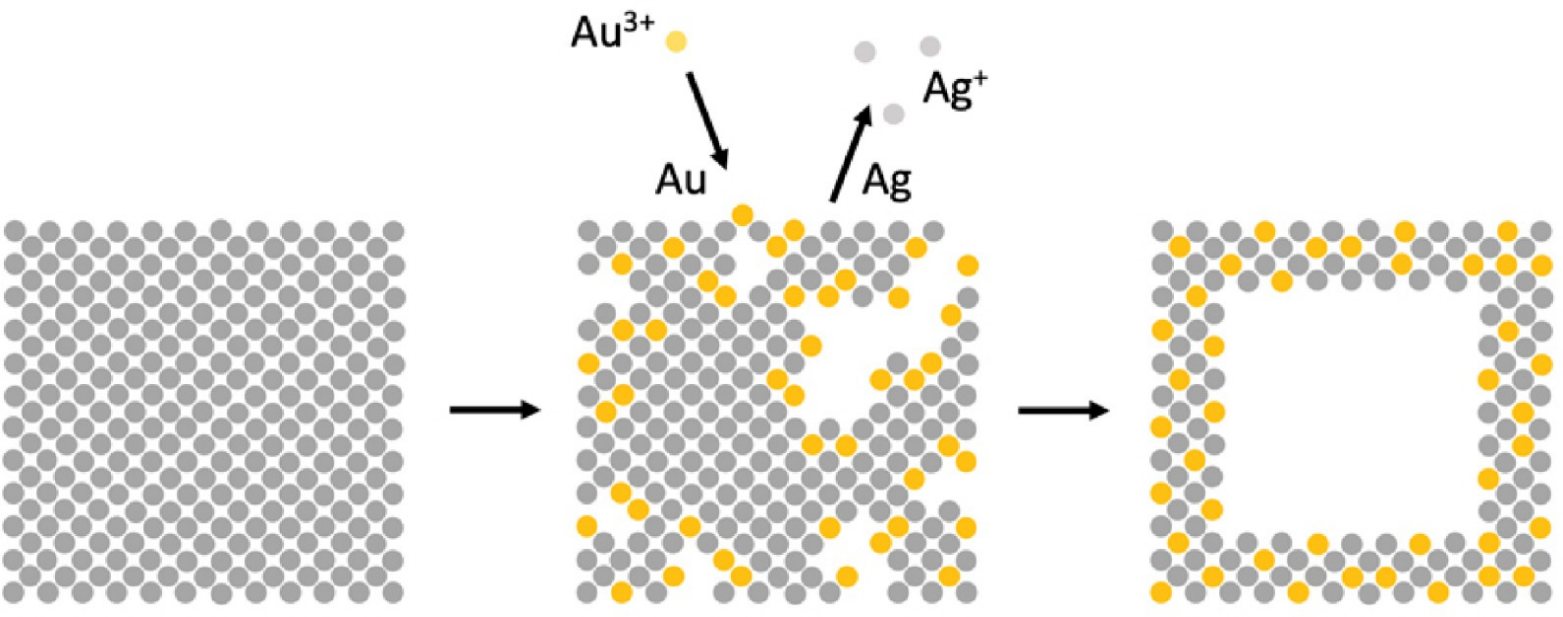
Schematic illustration of the galvanic replacement process taking place between a Ag nanocube and Au^3+^ ions at different stages.

Galvanic replacement is a well-known redox process that has already been discussed at length elsewhere in literature.^89^ However, its first observation in a nanoscale system on Ag nanocubes and continued relevance to Ag nanocubes warrants a brief discussion in this work.^27^ Despite some drawbacks discussed in the following section, galvanic replacement can still be a broadly useful synthetic method. In the case of hollow nanostructures such as nanoboxes and nanocages, galvanic replacement can be an extremely facile reaction, and in many cases, it only requires bringing a salt precursor into contact with the surface of another metal.^27^ Because Ag has a relatively low reduction potential of 0.79 V, it undergoes galvanic replacement reactions spontaneously with the salts of many other noble metals as shown in Figure 15.^66^ This has led to many reports on the synthesis of various hollow nanostructures.^104^ In general, there are a few basic principles that govern the final product obtained from a galvanic replacement reaction.^89^ The first is elemental composition, which can be controlled by adjusting the ratio of Ag nanocubes to the added salt precursor. If more than one salt precursor is used, the order of addition also becomes relevant.^105^ The second is internal structure, which dictates the dissolution and deposition sites of the two metals involved. This can be more pronounced in the case of multifaceted substrates, but may still affect substrates enclosed by a single type of facet, such as nanocubes, because of the difference in surface coverage by the capping agent at various sites.^78,106^ Finally, morphology is strongly dependent on the ability of the resultant metal to form an alloy with Ag.^68^ In the case of Ag–Au and Ag–Pd nanocages, the resultant surface was smooth because Ag and Au or Pd form a good alloy. In contrast, the Ag–Pt nanocages took on the general cubic shape but had a bumpy and irregular surface because Pt was much more prone to self-nucleation and island growth rather that interdiffusion. Controlling these three parameters, in addition to efforts to combine galvanic replacement with other processes (e.g., Kirkendall effect and coreduction) has led to a rich literature on numerous applications.^107−112^

### 4.7 Galvanic-Free Deposition

The sharp corners and edges on Ag nanocubes are often needed for LSPR, SERS, and other related applications. However, Ag nanocubes are prone to oxidation which can easily destroy these features. Therefore, it is pivotal to create bimetallic systems like Ag@Au core-frame nanocubes to protect the sharp corners and edges while maintaining the original optical properties of the Ag nanocube. Traditionally, galvanic replacement has been used to synthesize bimetallic structures with a hollow interior.^27^ This is because galvanic replacement is a spontaneous electrochemical process by which an already reduced metal is oxidized by the ions of another metal with a higher reduction potential. For example, a Ag nanocube can serve as a substrate onto which the Au ions can be reduced since Ag has a lower reduction potential than Au. However, because galvanic replacement facilitates redox reaction between two metals, the substrate metal is sacrificed when it is oxidized, leading to potentially undesirable results such as pitting, which tends to compromise the overall shape or morphology. To avoid this, we developed galvanic replacement free strategies for metal deposition on Ag nanocubes.^113^ Two strategies are discussed below, which leverage both the reduction and galvanic replacement rates.^114^

**Figure 16:**
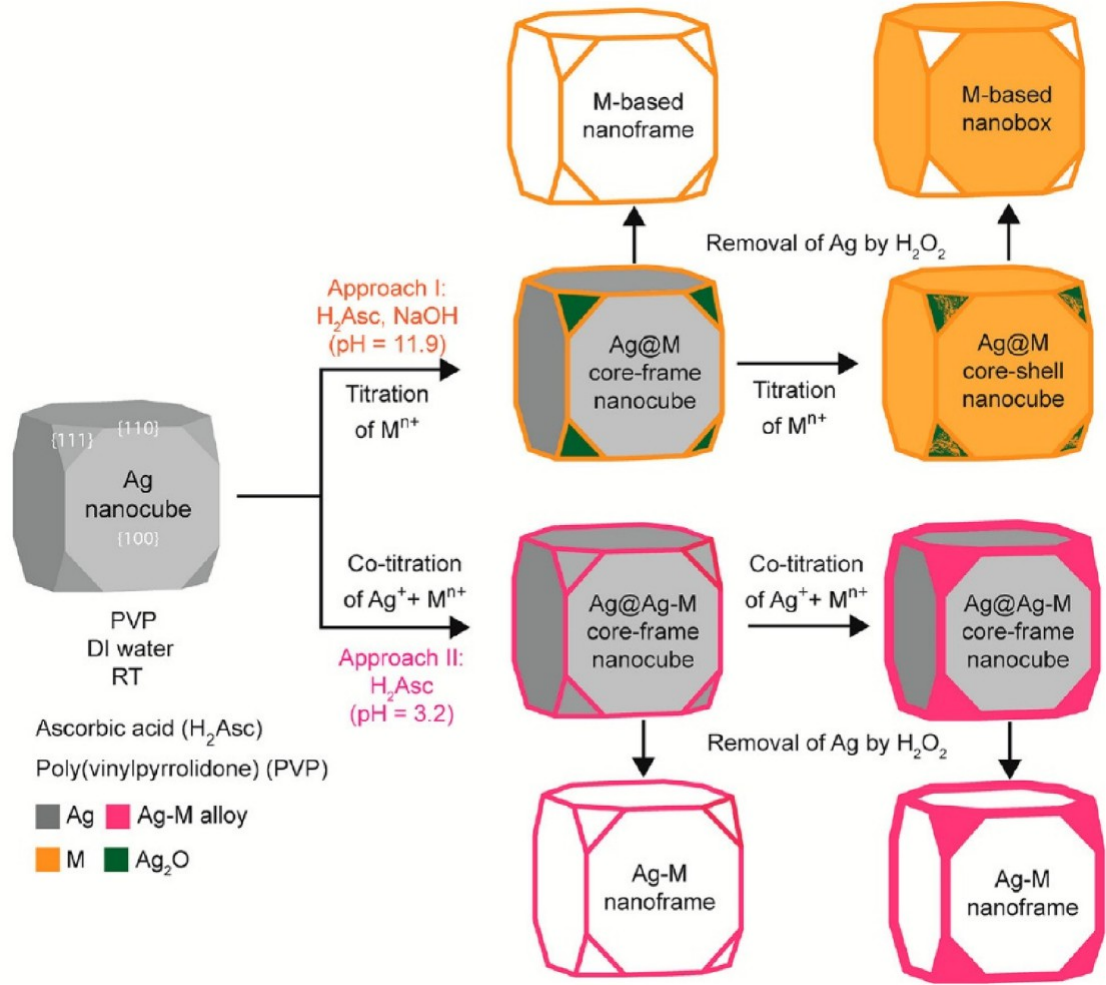
Schematic illustration of the two approaches to galvanic replacement free deposition of metals on Ag nanocubes and their resultant structures. Reproduced with permission from ref (114). Copyright 2017 American Chemical Society.

The first approach is shown in the top branch of Figure 16, which involves presynthesized Ag nanocubes, PVP, AA, and NaOH. The use of AA is uniquely important to this strategy because its reducing power is pH sensitive and one can readily access different reduction strength depending on the pH.^115^ In the pH range of 10.3–11.9, AA exists as the diascorbate ion, a significantly stronger reducing agent due to the negative charges on the two oxygens. Thus, the creation of an alkaline solution through the addition of NaOH ensures the reduction rate far outpaces the rate of galvanic replacement. This method is particularly effective in the creation of Ag@Au nanostructures although Ag and Au have a large difference in reduction potential.^113,116,117^ The HAuCl_4_ precursor is quickly reduced and deposited onto the edges, corners, and then side faces of Ag nanocubes. The thickness of the Au shells can be easily controlled by adjusting the amount of precursor added relative to the size/number of the Ag nanocubes used.^113^ The process can be pushed further to create either Au nanoframes or nanoboxes by controlling Au coverage and subsequently etching away the Ag nanocube core with 3% aqueous H_2_O_2_.^116,117^

The second approach is shown in the lower half of the schematic presented in Figure 16. In this case, presynthesized Ag nanocubes are mixed with PVP, AA, and Ag^+^ ions in addition to another salt precursor at a low pH. Because AA is a weak reducing agent at a pH of 3.2, this method relies on the addition of Ag^+^ ions to retard the galvanic replacement rate through Le Chatelier’s principle. However, because the addition of Ag^+^ creates alloyed frames, the possible resulting structures are limited in terms of composition. The creation of Ag@AuAg core-frame nanocubes requires the AgNO_3_ to HAuCl_4_ ratio to be at least 3 to prevent galvanic replacement.^118^ Metal precursors such as Na_2_PdCl_4_ and H_2_PtCl_6_, require much lower ratios because of the smaller difference in reduction potentials.^119,120^ Similarly, the alloyed component on the Ag nanocubes could be controlled by varying the amount of the salt precursor added. Etching the Ag core with aqueous H_2_O_2_ again resulted in nanoframes or nanocages, while nanoboxes could not be created because the Ag incorporated in the alloy also becomes etched, leaving a porous surface.

### 4.8 Catalysis

**Figure 17:**
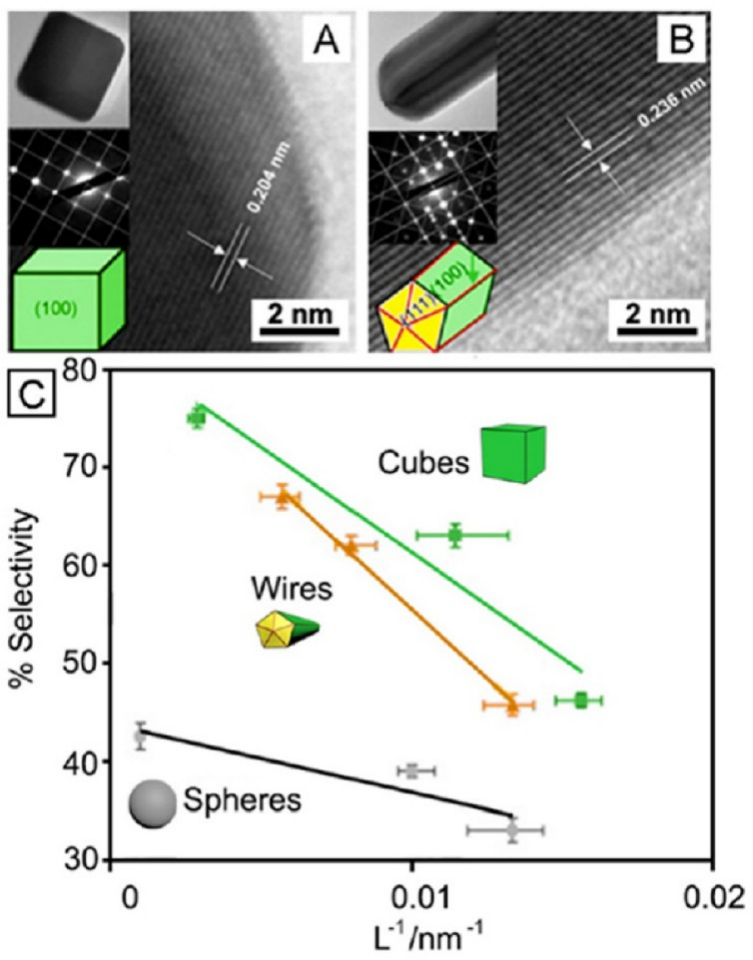
Catalysts based upon Ag for the ethylene epoxidation reaction. (A, B) High-resolution TEM images illustrating the surface structures of a Ag nanocube and Ag nanowire, respectively. Insets from top to bottom: TEM images of the nanocrystals at a low magnification; SAED patterns used for zone axis identification; and models. (C) Selectivity toward EO as a function of L^–1^ for cubes, wires, and spheres of three different sizes, where L^–1^ stands for the inverse characteristic length. Reproduced with permission from ref (125). Copyright 2010 John Wiley & Sons.

Ethylene oxide (EO) is an important industrial chemical that can be further converted to glycols, polyesters, or plastics.^121^ It can be obtained through oxidation of ethylene, with CO_2_ and H_2_O as the byproducts.^122^ Silver is particularly good at selectively oxidizing ethylene to EO by promoting the generation of a surface oxametallacycle (OMC) intermediate, which can then undergo isomerization reactions on Ag to generate EO.^123^ However, ethylene on the Ag surface still has the tendency to be overoxidized to undesired byproducts such as CO_2_ and H_2_O if the OMC intermediate is converted to acetaldehyde (AC) instead of EO.^121^ The traditional Ag catalysts prepared using the standard impregnation method were mainly covered by {111} facets, which show limited selectivity toward EO because the activation barriers to pathways involving EO and the undesirable combustion products are comparable.^124^ It is possible to enhance their selectivity toward EO by controlling the shape and size of the Ag catalytic particles. To this end, Linic and co-workers performed density functional theory (DFT) calculations and found that the reaction pathway toward EO was more favorable on the {100} facets than the {111} facets.^124^ This finding was confirmed by experimental studies, where Ag nanowires enclosed predominantly by the {100} facets were found to exhibit greater selectivity toward EO than conventional Ag catalysts. The same group later systematically investigated the effect of both size and shape on the selectivity by comparing Ag nanocubes, nanowires, and conventional catalysts with varied sizes.^125^ Specifically, Ag nanocubes exclusively covered by {100} facets showed the highest selectivity toward EO (Figure 17). Moreover, Ag nanocubes with larger sizes offered better selectivity, consistent with other studies. This is because the increase in size decreases the proportion of undercoordinated surface atoms at vertices and edges, which are responsible for diminished selectivity. Taken together, there is a need to identify the optimal particle size for this reaction because increasing size will compromise the mass-specific activity.

### 4.9 Integration of Catalysis and SERS

A particularly interesting intersection of applications arises when the optical properties of Ag nanocubes are combined with the catalytic capabilities of another metal.^126^ As briefly discussed above, the combination of Ag nanocubes with another metal, especially through a galvanic replacement-free synthesis, can lead to enhanced SERS activity by preserving sharp features and enhancing chemical stability of the underlying Ag structure.^113^ Significantly, these bimetallic particles can be utilized to monitor, *in situ*, the progression of a reaction for which one (or both) of the metals serves as a catalyst(s). The reduction of 4-nitrothiophenol (4-NTP), followed by the subsequent oxidation of 4-aminothiophenol (4-ATP), is the most commonly used model reaction to demonstrate the integration of SERS and catalysis because of the strong and distinct signals arising from each molecule.^127^ However, through careful observation of the SERS signals for these reactions, it has become clear that the actual pathways can vary depending on the specific metals utilized. In the case of Ag@SiO_2_/Au particles, SiO_2_ was deposited on the Ag nanocubes and then partially etched to create pores into which Au could then be deposited.^128^ This resulted in an “islands in the sea” configuration to ensure that the Au islands could not merge and remained small to boost catalytic activity. Observation of the SERS signals indicated that 4-NTP was first reduced by NaBH_4_ on the Au islands into 4-ATP and then oxidized by O_2_ on the exposed Ag to generate *trans*-4,4′-dimercaptoazobenzene (*trans*-DMAB) after 120 min into the reaction.

**Figure 18:**
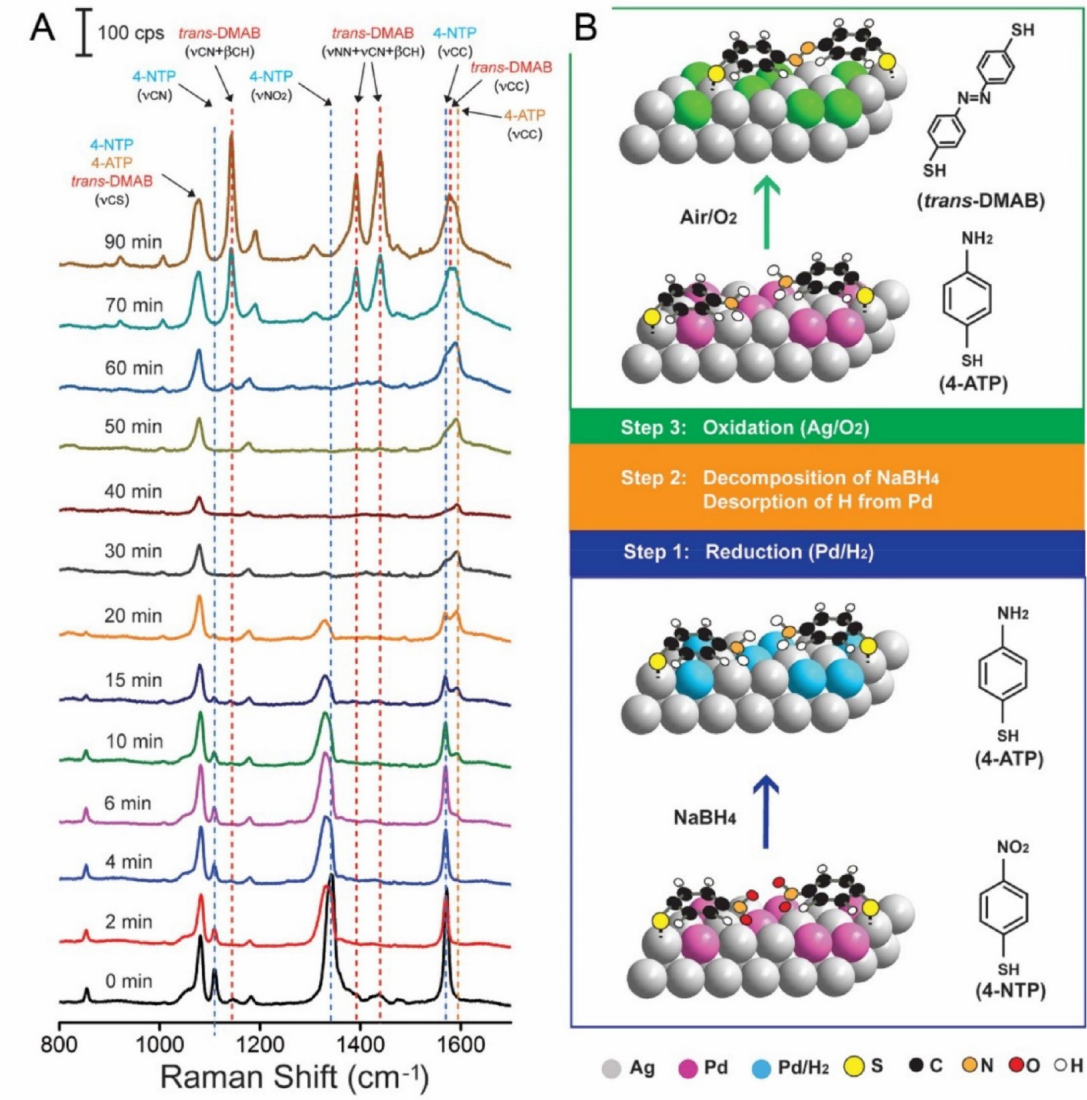
(A) Time-dependent SERS monitoring of the reaction starting from the reduction of 4-NTP by NaBH_4_ on Ag@Ag–Pd core-frame nanocubes with 2.2 wt % Pd. (B) Proposed reaction pathway for the Pd-catalyzed reduction of 4-NTP by NaBH_4_ and Ag-catalyzed oxidation of 4-ATP by O_2_ in the air. Reproduced with permission from ref (88). Copyright 2016 John Wiley & Sons.

In another study, HAuCl_4_ was titrated into a solution of Ag nanocubes in the presence of CTAC, creating Ag@Au–Ag core-frame nanocubes with slightly concave side faces.^110^ The concave side faces led to the presence of more undercoordinated Ag atoms while the sharper features resulted in an increase in both SERS and catalytic activity. In this case, 4-NTP was reduced to 4-ATP through the intermediate of *trans*-DMAB. It was suggested that this pathway is favorable when the adsorbed molecules could be oriented parallel to the Au surface.^129^ Similarly, it was discovered that Ag@Pd–Ag core-frame nanocubes also utilize this orientation.^88^ Typically, Pd and Pt are favored over Au as a catalyst for hydrogenation because they have high activities, but this would also make it difficult to capture intermediates to elucidate the reaction mechanism. The integration of catalysis and SERS has allowed for *in situ* analysis and careful characterization. For example, adjusting the Pd content of the nanoframe increased the rate of 4-NTP reduction to 4-ATP but did not affect the rate of 4-ATP oxidation to *trans*-DMAB, indicating that Ag is responsible for the activation of O_2_ molecules. The time-elapsed Raman spectra in Figure 18 clearly demonstrates this pathway. A few studies explore the integration of SERS and catalysis for other reactions. For example, the Ag@AgAu core-frame nanocubes fabricated by cotitration could be used to track the reduction of 4-nitrophenol (4-NP) to 4-aminophenol (4-AP) by NaBH_4_.^118^ Although the molecules are similar to 4-NTP and 4-ATP, the paper discussed the careful balance of Au content maintains a high catalytic rate but does not dampen the SERS signal. A more unique structure can be found in the Ag–Rh core-frame nanocubes.^130^ These particles were used to monitor the degradation of Rhodamine 6G (R6G) by catalytic reduction with NaBH_4_. At a 2.8 wt % of Rh, the R6G could be completely degraded within 8 min under clear SERS monitoring.

### 4.10 Hybridization with Semiconductors

**Figure 19:**
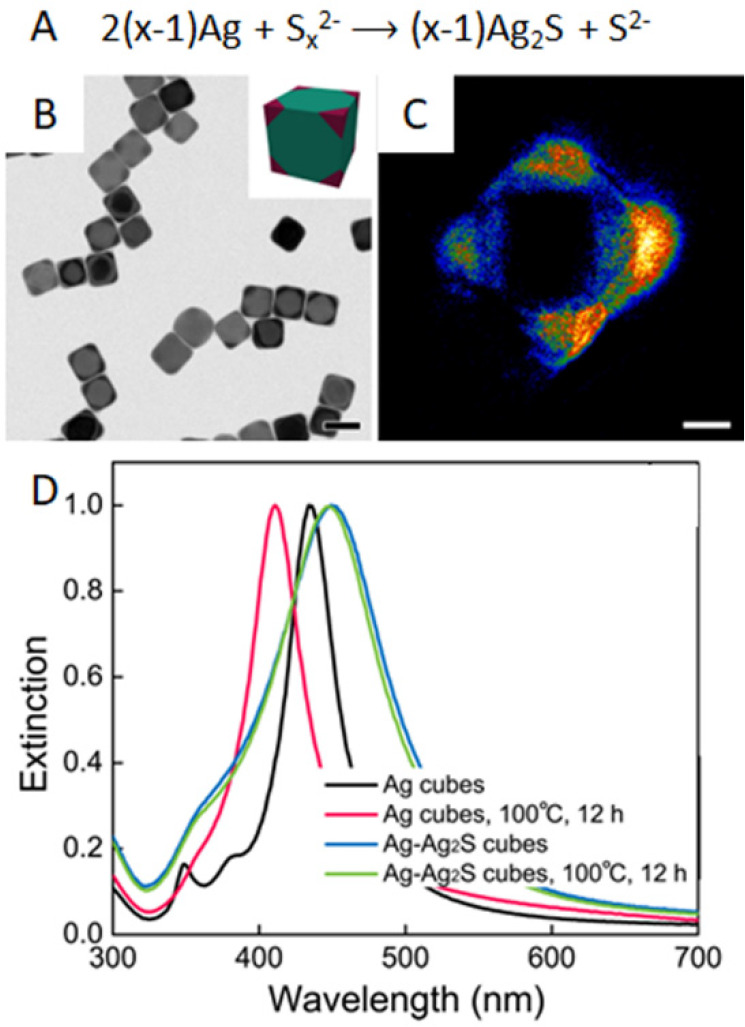
(A) Chemical reaction involved in the sulfidation of Ag nanocrystals. (B) TEM image of the product after sulfidation of the Ag nanocubes for 20 min, showing that the reaction preferentially occurred at all corners of the nanocube. The inset shows an illustration of the Ag nanocube with Ag_2_S formed at all corners. (C) Energy-filtered TEM image of a typical Ag–Ag_2_S hybrid nanocube. The scale bars are 50 nm in (B) and 10 nm in (C). (D) Extinction spectra recorded from aqueous suspensions of the Ag nanocubes and Ag–Ag_2_S nanocubes before and after the samples had been aged at 100 °C for 12 h. Reprinted with permission from ref (74). Copyright 2011 American Chemical Society.

Metal–semiconductor hybrid nanocrystals have attracted considerable interest due to their synergistic properties arising from the interaction between the individual components.^131−134^ Particularly, the hybrid nanocrystals enable the coupling of localized plasmon excitations generated in the metallic portion with the quantum confinement effects in the semiconductor portion, a feature vital to various plasmon-enhanced applications. Among Ag-based hybrid nanocrystals (e.g., Ag-ZnO and Ag–Ag_2_S), Ag–Ag_2_S is of particular interest because of its high optical absorption coefficient and chemical stability.^135−138^ Major efforts have been made toward the synthesis of Ag–Ag_2_S hybrid nanocrystals. While the majority of the nanocrystals possess a complete core–shell structure, it is also possible to selectively sulfurize different sites of a Ag nanocrystal for the fabrication of a core–satellite structure, as briefly discussed in Section 3.1.^74^ In a typical synthesis, an aqueous solution of polysulfide (Na_2_S_*x*_) was used to react with Ag nanocrystals (either Ag nanoplates or nanocubes) to generate Ag_2_S in the absence of O_2_, as illustrated in Figure 19. The sulfidation of Ag nanocrystals started exclusively from the corner sites because of the lower coordination numbers of Ag atoms and thus their higher reactivity. The Ag_2_S regions then grew at the expense of Ag. Unlike previous studies, the kinetics and degree of sulfidation could be easily and tightly controlled by varying the reaction time and/or the amount of Na_2_S_*x*_ added due to the larger size of S_*x*_^2–^ (relative to monomeric S and S^2–^ used in previous studies) and thus slower diffusion through the Ag lattice. By controlling the kinetics, the sulfidation could be confined exclusively to the corner sites of a Ag nanocrystal. The sharp corners play an even more important role than the strain induced between Ag and Ag_2_S regions, making the sulfidation always initiated at the sharp corners, instead of the interface created during the reaction.

The cubic shape of the Ag nanocubes was well preserved even though the crystal lattice experienced massive expansion during sulfidation. The lateral dimensions of the as-obtained Ag–Ag_2_S hybrid nanocrystal were much greater than those of the original Ag nanocrystals because of the higher molar volume of Ag_2_S relative to Ag. Significantly, the conversion of the corners from Ag to Ag_2_S helped prevent the nanocrystals from changing the shape during an aging process (e.g., at 100 °C for 12 h in the case of Ag nanocubes). According to the UV–vis extinction spectra recorded from the Ag and Ag–Ag_2_S samples, the in-plane dipole plasmon peak experienced a continuous red shift and depression with increasing fraction of Ag_2_S. In addition, the pattern of the plasmonic modes was also modified after sulfidation of the sharp corners of either Ag nanocubes or nanoplates. Although the higher dielectric constant of Ag_2_S was used to explain these changes in extinction spectra, this explanation was not supported by experimental observations. By combining both the theoretical and experimental studies on Ag–Ag_2_S nanoplates, Shahjamali and co-workers demonstrated that the proper model for the hybrid system described above might be a Ag@Ag_2_S core–shell structure, albeit the layer of Ag_2_S at the corner was much thicker than the Ag_2_S layers on the basal planes.^139^ It is the ultrathin Ag_2_S layers on the basal planes that contributed to the red shift of the plasmon peak. A similar scenario might also apply to the system involving Ag nanocubes.

## 5 Conclusion

Over the past two decades, significant progress has been made in the colloidal synthesis of Ag nanocubes and exploration of their fascinating optical and catalytic properties for a range of applications. A systematic investigation into their growth mechanism has shed light on the important factors that determine the geometric shape taken by the nanocrystals. The identified factors include the oxidative etching of twined species to ensure the formation of single-crystal seeds and the use of a proper caping agent to selectively passivate the {100} facets.^35^ The new discoveries allowed for the development of robust protocols for the facile synthesis of Ag nanocubes with exquisite control over both the shape and size. Compared to the counterparts made of other noble metals, Ag nanocubes exhibit excellent optical properties because Ag can support strong plasmon resonance over a broader range of wavelengths.^90^ This explains their widespread use in applications related to LSPR and SERS. In addition, combining the unique optical and catalytic properties of Ag also brings in more opportunities for applications, for example, photocatalysis and *in situ* probing of catalytic reactions through spectroscopic fingerprinting. For all these applications, the unique shape and thus sharp features on Ag nanocubes play an important role in augmenting their performance.

### 5.1 Future Challenges

**Figure 20:**
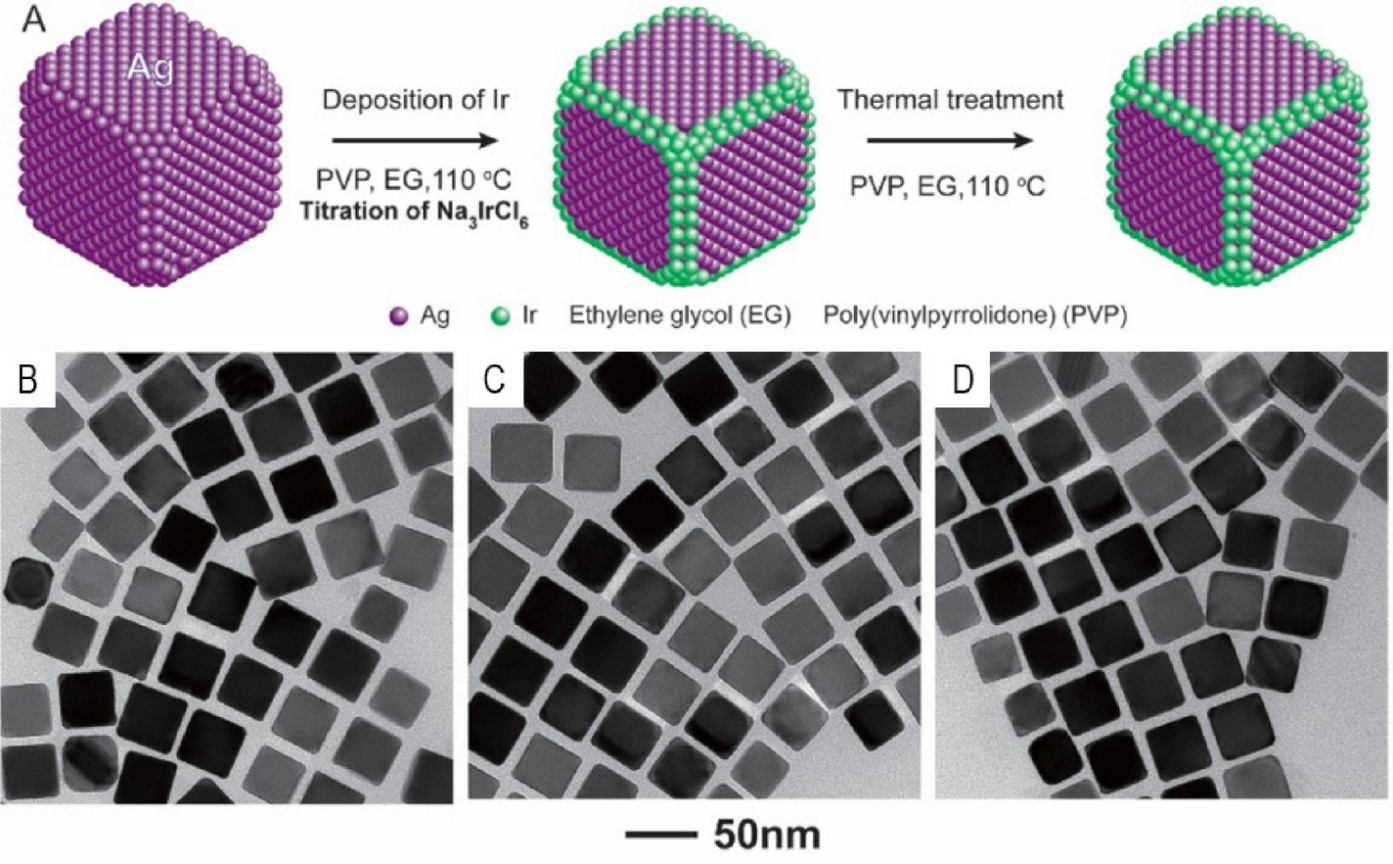
(A) Schematic illustration of the transformation of a Ag nanocube into a Ag–Ir core-frame nanocube by reacting with Na_3_IrCl_6_ in EG, followed by treatment in PVP/EG at 110 °C. (B–D) TEM images of the Ag–Ir nanocubes prepared by titrating 0.1 mL of the Na_3_IrCl_6_ solution (B) before and after thermal treatment in EG for (C) 10 min and (D) 30 min. Modified with permission from ref (142). Copyright 2020 Royal Society of Chemistry.

Despite the broad range of applications enabled by the success in colloidal synthesis of Ag nanocubes, many challenges still remain, especially in the context of shape preservation, long-term storage, and scaled-up production. The proneness of Ag to oxidative etching can be viewed as a disadvantage that may lead to easy dissolution in an oxidative, halide rich, or acidic environment.^86,90,140^ For example, the sharp corners and edges on Ag nanocubes are quickly truncated when aged in a polyol held at an elevated temperature in air.^141^ Such evolution into a rounded structure compromises their SERS activity due to the loss of hot spots for E-field enhancement. Additionally, the performance of Ag nanocubes as a catalyst toward selective ethylene epoxidation also deteriorates because of the increased proportion of {111} facets on the surface.^124^ People have started to address this shape instability issue.^141^ For example, we recently demonstrated that the overall shape of Ag nanocubes could be well preserved by passivating the most susceptible sites (i.e., corners and edges) with a more inert metal such as Ir (Figure 20).^142^ In a typical synthesis, Na_3_IrCl_6_ solution in EG was titrated into a suspension of Ag nanocubes in an EG solution containing PVP held at 110 °C. The derived Ir atoms were preferentially deposited onto the edges and then corners, generating Ag–Ir core-frame nanocubes. Stability tests indicated that the very small numbers of Ir atoms deposited on the edges and corners were able to prevent the Ag nanocubes from being rounded when heated in a PVP/EG solution under ambient pressure up to 110 °C. The Ag–Ir core-frame nanocubes were also found to embrace plasmonic and SERS properties comparable to those of the original Ag nanocubes.

### 5.2 Perspectives

Both the quantity and quality of Ag nanocubes need to be improved if this nanomaterial is to be seriously considered for use in industrial applications. In an early attempt, we have successfully increased the quantity of Ag nanocubes by 100-fold from 0.01 to 0.1 g for each run of the synthesis by modifying the NaHS-mediated polyol synthesis.^47^ Significantly, the 40 nm Ag nanocubes synthesized in a typical batch of the sample would be enough for the production of Au-based nanocages adequate for *in vivo* tests, including both optical imaging and photothermal cancer treatment, with at least 100 mice. It was of critical importance to ensure fast reduction by tightly managing oxidative etching involved in the reaction for the successful operation of this synthesis. The oxidative etching could be successfully eliminated by protecting the system with a flow of Ar gas. In parallel, it has also been reported that the production of Ag nanocubes could be scaled up by switching from the traditional batch reactor to a continuous flow reactor.^143−145^ It is expected that all these efforts will eventually push colloidal Ag nanocubes to a higher level of success in terms of both fundamental studies and industrial applications.
